# Sensory Changes and *Listeria monocytogenes* Behavior in Sliced Cured Pork Loins during Extended Storage

**DOI:** 10.3390/foods9050621

**Published:** 2020-05-12

**Authors:** Rita Silva, Jorge Pereira, Margarida Rouxinol, Luis Patarata

**Affiliations:** 1 Escola de Ciências Agrárias e Veterinárias (ECAV), Universidade de Trás-os-Montes e Alto Douro (UTAD), Quinta dos Prados, 5000-081 Vila Real, Portugal; rita.oliveiraesilva@gmail.com; 2 Campus da Penha, Estrada da Penha, Universidade do Algarve, Instituto Superior de Engenharia, 8005-139 Faro, Portugal; japer@ualg.pt; 3 MED—Mediterranean Institute for Agriculture, Environment and Development, Instituto Superior de Engenharia, Universidade do Algarve, Campus da Penha, 8005-139 Faro, Portugal; 4 Irmãos Monteiro S.A., 3830-527 Aveiro, Portugal; margaridacarmo@irmaosmonteiro.pt; 5 CECAV—Center of Studies in Animal and Veterinary Science, 5000-081 Vila Real, Portugal

**Keywords:** shelf life, cured pork loins, spoilage, sensory, CATA, consumer test, *salpicão*, meat products, challenge test, *Listeria monocytogenes*

## Abstract

Cured pork loins are sausages with a production tradition in several regions worldwide. They are made from one of the noblest cuts of pork, and for this reason cured loins are one of the most expensive pork meat products. Establishing the correct shelf life allows products to be accepted by the consumer, and to avoid the costs associated with shorter shelf lives. The aim of this study is: (1) to establish proper shelf life by evaluating the willingness of participants to consume and the sensory modifications that occur during prolonged storage via Check All That Apply (CATA) questions; and (2) to study the behavior of *Listeria monocytogenes* through a microbial challenge test. Sliced cured pork loins can be stored at 6 ± 1 °C for 105 days while maintaining a consumer acceptance of more than 75%. The freshness loss was associated mainly with a decrease in aromatic notes (particularly the smoke and cured aroma), and with the appearance of spoiled characteristics, specifically a sour/vinegar aroma and acidic taste that were detected by a reduced proportion of participants. The freshness evaluation was positively influenced by the typical characteristics of cured products, such as color and a garlic and wine aroma. Sour/vinegar aroma and acidic taste were the attributes most associated with higher freshness penalization. During the period of the test, *Listeria monocytogenes* inoculated onto the cured loin slices did not grow.

## 1. Introduction

Cured pork loins are different according to the production region. Variations in seasoning, size, cut (either longitudinal or transversal) and smoking determine the differences. All of these products are cured using salt and/or nitrate, and are dried to achieve a sliceable texture and an activity of water (aw) reduced enough to contribute to the safety and preservation capacity of the product [1,2,3,4,5]. 

Cured loins are made from one of the noblest cuts of pork and are, therefore, one of the most expensive pork meat products [6]. They usually have limited amounts of fat, which pleases the consumer concerned with health [7]. There are several differences between the traditional recipes used to prepare cured loins and industrial production, namely the use of strategies to improve control over foodborne pathogens and to deal with meat with low amounts of fat that dry very easily. To ensure the control of pathogens, the use of nitrite contributes to the inhibition of *Clostridium botulinum* and *Listeria monocytogenes*, among others [8,9]. The problem associated with non-spore forming pathogens with low infective doses like *Salmonella* or enteropathogenic *Escherichia coli* is commonly overcome by the use of high microbial quality raw materials and application of mild heat treatments at the beginning of the drying [10,11]. When dry-cured loins are smoked, the mild heat treatment is done during the smoking phase, applying a temperature between 55 and 65 °C during a period long enough to allow a reduction in the non-spore pathogens that could possibly be present [12,13,14]. Recommendations have been made to guarantee a reduction of 5D in the *Salmonella* population resulting from the processing. The hurdles used to achieve that reduction might include chemical preservatives, mild heat treatments, and drying [15]. Increases in demand for easy-to-use meat products and the opportunity to purchase smaller portions [16] has been driving the industry to introduce a final step of slicing the cured loins and packaging smaller portions. Slicing is always a sensitive step in the industry. Once sliced, cured loins are more prone to spoilage through chemical modifications, microbial growth, or the interaction between the two [17]. Chemical spoilage of cured loins includes lipid oxidation and pigment modifications, with consequences to the aroma and the color. To control rancidity, the manufacturer uses antioxidants and ingredients with antioxidant proprieties and packaging without oxygen [18]. The stability of pigments is highly dependent on the use of nitrite [19]. Another problem that might arise in packaged meat products is aroma loss during storage. This might occur as a result of the chemical modification of several aromatic molecules, or by scalping with the packaging material [20,21]. In these cases, the problem is not the occurrence of unpleasant aromas or aspect modifications, but aroma loss, or a slight aroma modification, due to the decrease in specific molecules with specific aromatic notes [22,23].

Despite the very high hygiene measures used in slicing, any accidental contamination with foodborne pathogens might result in a lack of food safety. It is of particular concern if the contamination occurs with *L. monocytogenes* or other psychotropic pathogens and if the product has intrinsic parameters, namely aw and pH, that allows their multiplication [24,25]. Taking into consideration the number of variables that might compromise sliced cured loin quality, determining shelf life is a task that should first guarantee safety, then retard spoilage mechanisms in order to maintain the desirable sensory characteristics (and therefore the acceptability of the product) [26]. When safety of the product is assured, as expected in an industry that uses the Hazard Analysis and Critical Control Points (HACCP) methodology correctly, the remaining problem is how long the deterioration modifications remain unnoticed by the consumer [27]. There are several strategies to evaluate shelf life, the most direct being to present sliced cured loins with different storage durations (and therefore with different deterioration levels) and ask consumers if they are willing to consume them, having considered their freshness [28,29]. For general market products, it is usually assumed that shelf life can be extended until the moment when 50% of consumers reject it. This limit considers that near the end of shelf life there are only a residual number of packages on supermarket shelves, and the risk of having half the consumers of these last packages unsatisfied is the risk taken when considering the alternative—the price of a shorter shelf life. When defining the shelf life of an expensive product, it might be wise to reduce the rejection limit to a smaller amount of consumers (e.g., 25% or 10%) when considering the costs of that reduction [29,30].

The aim of the present study was: (1) to establish proper shelf life by evaluating the willingness of consumers to consume and the sensory modifications the occur during prolonged storage via a Check All That Apply (CATA) test; and (2) to study the behavior of *L. monocytogenes* through a microbial challenge test.

## 2. Materials and Methods

### 2.1. Aging Test

#### 2.1.1. Samples 

Cured pork loins, locally named salpição, were obtained from an industrial unit on the week of their production. The cured loins were produced using the normal industrial manufacturing process. White commercial crossbreed pork carcasses (80 to 90 kg) of both sexes were obtained two days after slaughter. The loins were excised during the carcass dressing. Cured pork loins were prepared by cutting the loin transversely in single pieces about 5-cm thick and weighing about 400 g, then seasoning with red wine, salt, dextrose, garlic, and spice extracts. Non-meat ingredients were also included, specifically soya protein concentrate, milk powder, pork hemoglobin, and the food additives sodium nitrite, trisodium citrate, and pentasodium triphosphate, according to the limits of addition previewed in the Regulation UE 1129 [31]. The mixture was inoculated with a commercial Lactic Acid Bacteria (LAB) starter culture to prevent the growth of unwanted microorganisms during the resting period and the beginning of the smoking. After 48 h of resting at 4 ± 2 °C, pieces of loin were stuffed in collagen casings, tied, and hot-smoked until they achieved an internal temperature of 65 °C. The cured loins were then dried until they achieved a water activity of 0.93. Finished products were sliced (±2 mm). The slices were pooled and distributed in approximately 10 slices/package, corresponding to around 150 g of product, then vacuum packaged. Once a product is made using normal industrial conditions, there is no possible link between one cured loin and the slices in a package. The slices could come from the same cured loin or several different ones. The packages were composed of different films in the lower and upper part; both were coextruded films of polyamide and polyethylene (PA/PE) (Termofilm, Famalicão). The characteristics of the films were: upper part—0.17 mm, 167.50 g/m^2^, water vapor transmission < 3 g/m^2^.d, and oxygen permeability < 25 cm^3^/m^2^.d.bar; lower part—0.12 mm, 118.00 g/m^2^, water vapor transmission < 3 g/m^2^·d, and oxygen permeability < 25 cm^3^/m^2^.d.bar. The upper film was 0.17 mm, 167.50 g/m^2^, water vapor transmission < 2 g/m^2^·d, and oxygen permeability ≤ 30 cm^3^/m^2^.d.bar.

The sampling procedure used in the present experiment is presented in Table 1. Once the products had a strict standardized manufacturing process with a high level of repeatability between production batches, we used samples from two different batches produced in consecutive weeks: one still in the beginning of the shelf life (6 packages), and one at the end of shelf life (9 packages). These samples were used in focus group (FG) interviews to gather vocabulary associated with the spoilage of cured loins and to evaluate the eventual effects of freezing on the sensory characteristics of the products. For each of the three FGs, one package of samples was used from the beginning of shelf life, one was used from the end of shelf life and one was stored at 30 °C during 4 days to force the spoilage and allow the participants to identify the characteristics associated with spoilage. To estimate whether freezing influences sensory characteristics, one set of three packages of freshly obtained samples and another set at the end of shelf life were frozen and thawed. The samples not frozen were the same used for gathering vocabulary. 

For the consumer test, 90 packages were transported to the laboratory under refrigeration, then divided into seven groups and stored at 6 ± 1 °C for a total of 126 days, corresponding to seven storage periods with expirations spaced 21 days apart (i.e., at 0, 21, 42, 63, 84, 105 and 126 days). Upon expiration of the stipulated period, the samples were frozen to −18 °C until sensory evaluation, to allow a reverse storage approach. This approach enabled us to analyze samples with different storage durations simultaneously [27]. Before sensory analysis, samples were thawed at 6 ± 1 °C for 48 h. A similar scheme of storage was used for microbiological and physical-chemical analysis, but the analyses were performed immediately when the storage duration expired. Once these parameters were not directly related to the aim of the work, these analyses were made for every other time period, plus the 63 days that corresponded to the shelf life established by the manufacturer. The analyses were made in triplicate, with each one corresponding to one package according to the scheme presented in Table 1. After collecting the samples for microbiological analysis, the remainders of the packages were used for color evaluation, pH, and water activity. 

The challenge test was made using samples from the two batches. Once they arrived at the laboratory with one week of difference, we kept the samples from the first batch at 2 °C for one week. The packages were opened in aseptic conditions, and the slices were distributed for new packages, with five slices per package (Table 1).

#### 2.1.2. Sensory Analysis

Focus groups (FGs) were used to evaluate the effect of freezing on the sensory characteristics of the cured loins and validate the usefulness of the reverse storage strategy used, as well as to identify vocabulary with the potential to describe the freshness and spoilage of cured pork loins [32]. Three focus groups were carried out: one with six participants (three women, 25 to 62 years) who had previous experience related to the sensory analysis of cured meat products, and two with naïf consumers with 10 (seven women, 21 to 35 years) and eight elements (five women, 18 to 24 years). All the FG interviews took place in the same city in the north of Portugal. The sessions were carried out in a brightly colored room with artificial illumination, and were conducted by a researcher with previous experience with consumer FGs [33,34]. A moderator explained the operation and objectives of the FG, and the identifications of participants was recorded. In the first part of the FG, participants evaluated frozen-thawed samples (and not frozen samples) that were presented simultaneously and identified by random three-digit codes. Participants were asked to observe, smell, and taste the samples, to indicate if they perceived any difference among the samples, and to point out the characteristics that they found most perceptible in each sample. In the second part of the FG, freshly obtained, end-of-shelf-life, and temperature-abuse-stored samples were presented. A discussion was conducted to identify the characteristics, particularly regarding aspects and aroma, that participants detected in the samples. The moderator insisted that the participants should focus on freshness/spoilage characteristics. At the end of the session, the moderator reviewed the main characteristics gathered during the discussion with the group. The sessions were audio-recorded and further transcribed to inventory the vocabulary generated. 

Consumer test was performed with total of 81 consumers. They were recruited from the professional and personal relationships of the researchers. Less than 20% of the participants were students. The group was composed of 58% women, and the mean age was 39 ± 15 years (21–65). Of these participants, 90% were regular consumers of dry-cured meat products, while the reaming 10% were sporadic consumers. Those not liking cured sliced loins did not accept the invitation. It was not possible to conduct this work with a randomly selected group of consumers that might represent a more appropriate approach. Nonetheless, the group of consumers represented several socio-demographic groups and were consumers of the product. On the other hand, none of the participants had any relationship with the manufacturer or the meat products industry, and most of the respondents were not connected to food science, aspects that are usually pointed out as being potential biases of a group [35,36].

The test was divided into two sessions with the same participants to avoid the overload of analyzing seven samples in the same session. Storage durations were distributed for the two sessions using alternate times (Table 1). The presentation of samples was made with three (21, 63, 105 days) or four samples (0, 42, 84, 126 days) in the same session, which were identified using a random three-digit number registered in a printed form. To avoid an order effect bias, five different orderings of the samples were provided, and participants were asked to follow the testing order according to each version. The samples were presented individually in a white dish with their number. All dishes were arranged previously in the testing booth and covered with white paper. Each dish contained two half-slices, one from each batch. The packages were opened 30–40 min before the test and maintained in the preparation room of the sensory analysis laboratory (18 ± 2 °C). The tests were conducted in individual white booths under artificial illumination. Crackers and spring water were made available to participants. 

For each sample, participants were asked to rate its freshness using a five-point scale (where 1 corresponded to “not fresh” and 5 corresponded to “very fresh”) and to indicate their consuming and purchasing intentions using a binary yes/no question. They were also asked to fill in the CATA part of the form, which consisted of a list of 24 attributes (9 for aspects, 11 for aroma, and 4 for taste—see Table 2 and related text) considered relevant to each sample.

#### 2.1.3. pH, Water Activity and Instrumental Color Measurement

Cured loin pH was measured directly in minced samples with pH m (model MicropH 2002, Crison, Barcelona, Spain). Water activity was measured in a Rotronic Hygroscope DT apparatus with a WA40 probe (Bassersdorf, Switzerland). Color was measured directly in the slices 30 min after opening the packages via a tristimulus color analyzer Minolta CR 310 (Minolta, Osaka, Japan) with a standard illuminant D_65_ using L*a*b* color space.

#### 2.1.4. Microbial Analysis

Microbiological analysis was performed at days 0, 42, 63, 84, and 126 of storage. After opening each of the three packages considered for each storage duration, 10 g was randomly cut from at least three slices then weighed and suspended in 90 mL of peptone water, as described in the reference [37], and analyzed LAB, mold and yeasts, and *Enterobacteriaceae* and *Pseudomonas* spp. LAB counts were determined on De Man, Rogosa and Sharpe Agar and incubated under at 30 °C for 2–3 days; mold and yeasts were determined on Rose-Bengal Chloramphenicol Agar Base and incubated at 25 °C for 5 days; *Enterobacteriaceae* were determined on Violet Red Bile Glucose Agar and incubated at 37 °C for 24 h; and *Pseudomonas* spp. were determined on Pseudomonads Agar Base with Cetrimide, Fucidin and Cephalosporin supplements and incubated at 25 °C for 48 h. All culture media were from Biokar (Allonne, France). Results are expressed as log cfu/g. For statistical purposes, when the microorganism count was below the detection limit, it was considered to be zero. Estimated counts were considered for data analysis when countable colonies were present but below the countable range.

### 2.2. Challenge Test with Listeria monocytogenes 

A challenge test was performed to study the behavior of *L. monocytogenes* during the storage of sliced cured pork loins, as described in the literature [16]. Freshly manufactured samples were obtained from the industry and were contaminated with a mixture of four strains of the pathogen: one strain from a culture collection (ATCC 35152), and three strains previously isolated from meat product industrial environments (laboratory collection). The cured loin slices were contaminated with 0.1 mL of the pathogen mixture to achieve around 3 log cfu/g. After 30 min of resting, slices were vacuum packaged in the same material indicated in Section 2.1.1. Packaged samples were stored at 6 ± 1 °C. The count of *L. monocytogenes* was performed immediately after packaging, then again at 42, 63, 84, and 126 days. For each storage duration, three packages were reviewed to perform the *L. monocytogenes* count. For each sample, slices were unpacked, weighed, and homogenized with the necessary volume of NaCl 0.85% to achieve an initial dilution of 0.1 g/mL. Serial decimal dilutions in NaCl 0.85% were prepared, and 0.1 mL of appropriate dilutions was spread onto selective Oxford Agar (Biokar BK110+ BS003, Biokar Diagnostics, Beauvais, France) followed by incubation at 30 °C for 24 to 48 h. When low counts were expected, 0.5 mL of the initial dilution was inoculated on two Petri dishes (0.25 mL each), dried in the laminar flow (to avoid biofilm formation), and counted as the total colonies in both dishes. Results are expressed in log cfu/g.

### 2.3. Data Analysis

Freshness evaluations were analyzed using one-way ANOVA to compare storage durations, with participants considered a random effect [36]. The comparisons of microbial counts—expressed in log cfu/g, instrumental color parameters, pH, and *L. monocytogenes* counts—were made using ANOVA for the three packages analyzed for each storage duration (Table 1). A Tukey test was used to determine significant differences (*p* < 0.05). An evaluation of freshness was compared between the consumption and purchasing intentions (yes/no using a *t*-test). The Pearson’s correlation between the LAB counts (expressed in log cfu/g) and pH was computed. The CATA results were recorded as 1 when a participant checked an attribute and 0 when they did not. The data were analyzed by factor analysis. Each attribute was compared between formulations using a Cochran test. The mean impact of each CATA attribute in the freshness evaluation was computed. The impact of each CATA attribute on the freshness evaluation was calculated only for the longest storage duration, when the spoilage notes were more easily detected. All the statistics were calculated using XLStat (Addinsoft, Paris, France). Raw data might be accessed at http://www.mdpi.com/2304-8158/9/5/621/s1 (Supplementary Materials).

## 3. Results and Discussion

### 3.1. Aging Test

**Figure 1:**
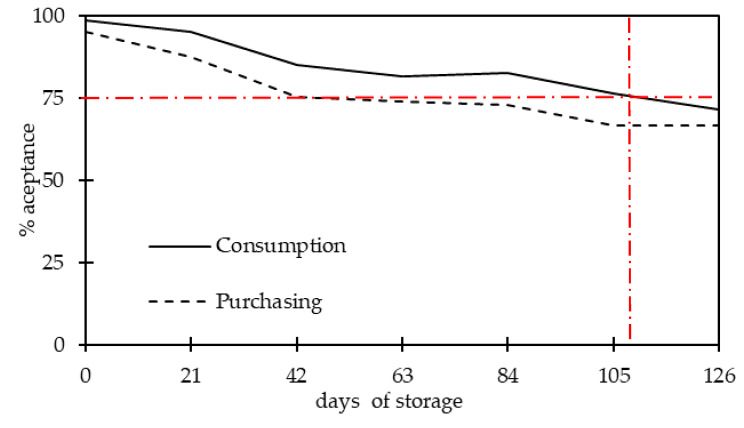
Consumption intention (continuous line) of sliced cured pork loins stored at 6 ± 1 °C for 126 days according to 81 participants. Purchasing intention (dashed line).

**Figure 2:**
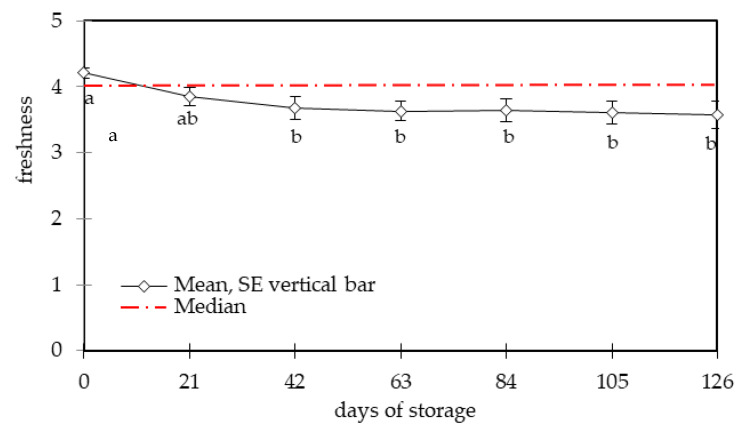
Assessment of the freshness of cured loins stored at 6 °C for 126 days; a, b (continuous line) followed by different letters are significant differences *(p* < 0.05); bars indicate the standard error for each assessed time. The dashed red line indicates the median values.

The sliced cured pork loins used in the present work had an aw of 0.93 and a pH of 5.8. A product with these stability parameters should be stored under refrigeration [7], which the manufacturer recommends. This product had a shelf life established by the producer of two months. To evaluate the possibility of extending shelf life, we doubled the storage period and evaluated consumer acceptability via the consumption intention for each storage duration (Figure 1). Purchasing information is presented as accessory information once the sensory shelf life had been determined based on consumption intention [30,38]. An evaluation of freshness was also performed (Figure 2). 

The consumption and purchase intention followed a similar trend during storage, with the purchasing intention being slightly lower than the consumption intention. This occurred as a result of participants electing to consume the product from the perspective of its freshness, but indicating that they were not willing to purchase it because it was a type of cured loin that they do not usually purchase, among other factors (as reviewed in reference [39]). According to the information gathered from the FG interviews and informal information from the feedback of the participants at the end of the sensory tests, this type of cured loin was very industrial-standardized, which was not appreciated by the participants that had consumption habits associated with more traditional varieties of the product. The sliced cured pork loins showed a high preservation capacity, and were accepted until the longest storage duration (126 days) by most of the participants with a slight reduction of parameters. A 50% level of rejection among participants, which is typically used to define the end of the sensory shelf life for general products [40], was not achieved during the testing period. Cured pork loins are, together with cured ham, the most expensive meat product made by the pork transformation industry. Thus, the use of stricter criteria to establish the culmination of shelf life is recommended. With that under consideration, it might be recommended that the shelf life of foods be limited to the moment there is a sensory rejection of 25% (or 75% acceptance) [29,36,41]. Using this criterion, the shelf life of sliced cured loins would be limited to 105 days, as at the longest storage duration (126 d) the consumption intention was expressed by only 71.6% of participants, which is already below 75%. This cut-off point should be established so that, at the end of shelf life, only a small number of packages would still be available on supermarket shelves. Thus, the manufacturer assumes the risk of having 25% or less of consumers consider the product not fresh once they represent a residual number of individuals, which is a risk that is necessarily tied to the costs of shorter shelf life [28,29,41,42]. Consumption and purchasing intention were associated with an estimation of freshness, as can be inferred by the parallelism between the results in Figure 1 and Figure 2, which both had a similar slight slope. The mean values of consumer-accepted freshness (via consumption intention), considering all storage durations, was 3.97 ± 0.79, which is significantly higher (*p* < 0.001) than those not accepted by the participants (2.51 ± 1.73). A similar trend was observed for purchasing intention (accepted 4.04 ± 0.74; not accepted 2.71 ± 1.12; *p* < 0.001). Still, in the period of high acceptance, the freshness evaluation was statistically different *(p* < 0.05) only between the first day of storage and all the other storage durations. After the initial more accentuated slope, the freshness assessment was similar from the 42nd day of storage until the end. The median values (Figure 2) also reflect the uniformity of the freshness evaluation during storage.

**Figure 3:**
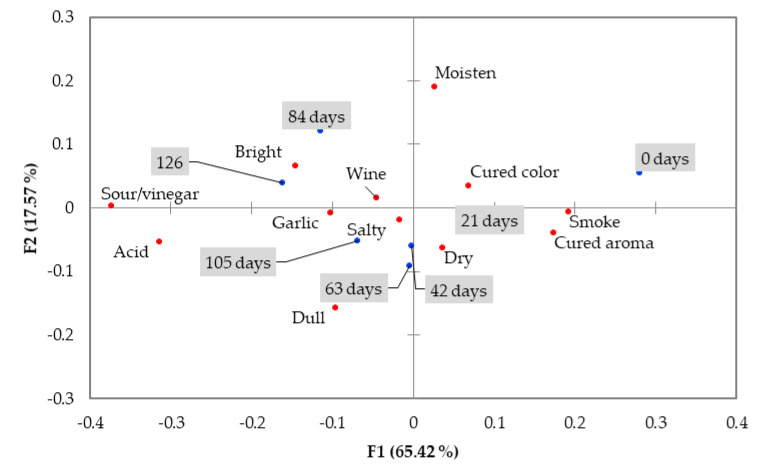
Attributes of cured loins and different storage durations tested (grey boxes) in the space defined by the first two factors.

To understand what modifications in the product were associated with a reduced consumption intention/freshness evaluation, participants were asked to identify those attributes of the cured loins (from a list provided) that they considered relevant at different storage durations. The results are illustrated in Figure 3. A statistical comparison of the frequency of checking each attribute for each storage duration is presented in Table 2. 

From the initial list of 24 attributes, 11 (slime, brownish color, darker spots, greenish spots, rancid, sulfur, mold, ammoniac/rotten, butter, piquant, sweet) were withdrawn from the analysis if they were used by less than 20% of participants for all the storage durations [43]. That strategy of not considering results checked by less than 20% of consumers is widely used [44,45,46,47]. Plaehn [48] justifies this from a practical manufacturer’s point of view that usually weighs risk versus revenue and profit margin. 

Even though the attributes “sour/vinegar” and “acid” had a frequency of use lower than 20% for most of the storage durations, they had frequencies or 20% or more at the longer durations, and so were kept. The first factor (F1) in Figure 3 is very discriminative of the freshness and spoilage attributes. On the right side of the plot are the variables associated with the fresher sliced cured loins (0 and 21 days of storage). This side of the plot includes the characteristic attributes of cured loins, with the higher factor loadings being smoke and cured aromas. The cured loins with longer storage durations (84, 105, and 126 days) are located in the left side of the plot, and are associated with the spoilage-related attributes “sour/vinegar” and “acid”. Curiously, two of the middle storage durations (42 and 63 days) were projected precisely on the origin of the F1, indicating a clear correlation between loss of smoke and cured aromas and accrued sour/vinegar aroma and acid taste. These four attributes were the only ones with significative differences (*p* < 0.05) between storage durations (Table 2). Still, for sour/vinegar aroma and acid taste it was not possible to locate differences between durations, probably because the significance level was close to the 0.05 limit and there was a reduced number of checks for those attributes. “Cured color” also suffered a reduction in frequency of checks by the participants, but it was not significant (*p* = 0.097).

The aspect characteristics were not different (*p* > 0.05) between any storage durations. The cured color, which seems to be discriminative in the factor analysis (Figure 3), had a tendency (*p* = 0.097) to be noted by fewer participants, from 79% at the beginning of the experiment to 61% at 126 days of storage. In the same sense, the color parameters a* and b* did not have differences among the tested storage durations (Table 3). The other CATA attributes studied—”moisten”, “dry”, “bright”, and “dull”—were expected to have a similar pattern to the one observed with the L* parameter, which decreased significantly from the middle of the storage duration. However, these CATA attributes were similar between all the storage durations, suggesting that the differences obtained by the instrumental evaluation of color were not significant enough to be perceived by the participants.

The results observed using the CATA approach indicate that, as the storage progressed, two phenomena contributed to the loss of freshness and reduction in consumption intention. On one hand, sliced cured loins lost their aromatic characteristics, while on the other hand, some spoilage characteristics became noticeable by some participants. The loss of aromatic characteristics might be associated with the interaction with the packaging polymer. Volatile molecules might be lost either by permeation or migration through the package or from sorption by the container [20]. The smoke aroma reduction was probably associated with the migration to the packaging material of some of the smoke molecules. The multilayer package used had a layer of polyethylene at its front that was in contact with the food, to facilitate the thermal sealing, and this is particularly prone to scalping organic molecules [22,49]. The study of polycyclic aromatic hydrocarbon (PAH) migration has mainly focused on reducing compounds with potential carcinogenic effects on humans [50,51,52]. However, these PAHs are also very aromatic [53,54], and their inclusion in the package probably affected the aroma of the sliced cured loins. The loss of typical aromatic notes may also be related to the chemical modifications of the compounds that form the loins’ aromatic fraction [55]. The cured aroma is highly related to the presence of several aldehydes that are formed from the catabolism of ramified amino acids. The 2- and 3-metil-butanal and the 2-metil-propanal formed from the catabolism of leucine, isoleucine, and valine are compounds present in cured and fermented meat products, and cured hams in particular, and have become associated with their typical aroma [56,57]. These compounds can be oxidized or reduced to their respective acids and alcohols, and they might have slightly different aromatic notes or, more importantly, different detection thresholds that could have a strong effect on aroma detection by consumers [58,59].

The formation of aromatic ketones due to lipid oxidation is further associated with the characteristic aroma of cured meat products [60], and in products made with wine, the consumer associates the cured aroma to the characteristic wine aroma [8]. The aromatic compounds formed during curing and those from the seasoning might experience chemical modifications during storage, resulting in different intensities or perceptions of the aroma [61]. Of the characteristics that were associated with spoilage, the sour/vinegar aroma and acid taste were the only notes associated with aged samples, which is a consequence of the fermentation of the carbohydrates by the LAB [62]. The increase in frequency of these notes being perceived by participants followed the same trend of the LAB count (Table 3), with higher references to a sour/vinegar aroma and acid taste when the counts of the LAB were already detectable (from the 42nd day of storage). That effect of LAB growth was also traduced in the pH evolution (*r* − 0.84, *p* < 0.05), which is a common occurrence since the production of lactic acid, and eventually acetic acid, is the main factor responsible for pH reductions in meat products that support the growth of LAB and incorporate carbohydrates into their formulation [63]. Cured loins were inoculated with an LAB starter culture (which can play an important role in controlling undesirable microorganisms during the resting phase) before smoking. Once the product was mild-heated there was a reduction in the LAB count, which was below the detection limit at 0 days. Among the non-spore forming microorganisms that survived the hot-smoking process, LAB and coagulase-negative cocci were among those with higher odds of dominating the product (due to its thermoduric nature [64]). During the storage, the surviving LAB began to grow and were counted at a low level (1.79 ± 0.73 log cfu/g) at 42 days of storage. These bacteria, due to their psychotropic nature and excellent adaptation to the cured loins ecosystem, dominate the product’s microbiota, which contributes to avoid the multiplication of unwanted microorganisms, pathogens, or responsibility for spoilage [25,65]. In certain fermented meat products, the production of diacetyl by the LAB involved in the fermentation process resulted in products with buttery and sweet aromatic notes. Diacetyl is produced particularly by *Brochothrix thermosphacta*, which can dominate the microbiota of packaged thermal processed meat products [66]. In the cured loins in the present study, the dominant LAB did not demonstrate this activity, since the buttery aroma was only noted by a residual number of participants (butter < 4%; sweetish < 15%). In meat products, the multiplication of *Enterobacteriaceae* and *Pseudomonas* are frequently associated with the occurrence of pungent spoilage odors due to strong amino acid catabolic activity [27]. In the sliced cured loins, the counts of these two groups of microorganisms were below the detection limit during the full aging period. The results from the CATA analysis were in accordance with the microbial counts, and the aromatic notes usually associated with amino acid catabolism, ammoniac/rotten, and sulfur were noted only by a residual number of participants (<2%). A slight increase in molds and yeasts was observed for the longest storage periods. The count of these microorganisms was made in Chloramphenicol Rose Bengal Agar, and we registered only the total number of colonies. The samples were vacuum packaged and, thus, the counts were expected to be mostly yeasts, as yeasts can grow in the absence of oxygen and under the restringing conditions of cured meat products [67,68,69,70]. Moreover, when the packages were opened for microbial analysis or sensory analysis, no mold growth was ever detected on the surfaces of the sausages. The counts of these microorganisms were 2.69 ± 0.61 log cfu/g at 84 days and 3.14 ± 0.79 log cfu/g at 126 days. Since molds are aerobes and yeasts growth faster in the presence of oxygen, their slow growth might be associated with a competition with the LAB, which were always in a higher number. Mold aroma is usually associated with the severe growth of molds on the surface of a product [71]. In the present study, the low amount of yeasts and molds had no implications on sensory characteristics, since that attribute was seldom used (<et 4%) by participants in the CATA test. Theoretically, the growth of yeast could have had implications on aroma if these microorganisms were associated with chemical modifications, resulting in aromatic molecules (namely, esters and alcohols that impart fruity notes to the product [72,73])—but that was not the case. Yeast can produce acetic acid, which could increase the sour/vinegar aroma perceived mainly in the longest storage durations. However, the reports on the effect of yeasts on meat product aroma concluded that only when yeasts have counts of 6 log cfu/g is their activity is sensorially detected [74], and its effect in the packaged sliced cured loins was probably marginal, as the aroma variable of sour/vinegar and acid taste were noted only by a small proportion of participants.

Cured pork loins usually have a low amount of fat. Combining that reduced fat with vacuum packaging and the presence of food additives that are not classified as antioxidants but have a role in preventing lipid oxidation (e.g., sodium nitrite, trisodium citrate, and pentasodium triphosphate), it was not assumed that the sliced cured loins experienced lipid oxidation. However, this attribute was maintained for the CATA test, as the initiation of oxidation might occur in some products before packaging, and the further modification can occur, albeit slowly, during long storage periods [18,75]. As observed for other spoilage aromatic notes, rancidity was residually noted (<4%). 

**Figure 4:**
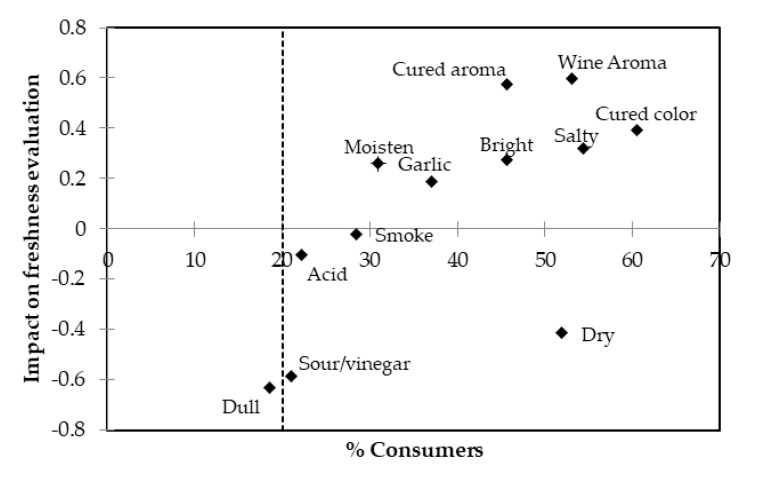
Mean impact on the freshness evaluation of the attributes checked by the participants at 126 days of storage. The dashed vertical line defines 20% of participants.

The CATA analysis allowed us to understand the loss of freshness of sliced cured loins during storage. The impact each attribute had on the freshness evaluation was assessed only using the data of the longest storage duration (126 days) (Figure 4).

In Figure 4 the impact that each characteristic had on the freshness evaluation in the five-point scale is plotted. As previously pointed out, a sour/vinegar aroma and acid taste were the two main sensory traits associated with the spoilage process. These attributes were noted by 21% and 22% of participants, respectively, for the longest storage duration (126 days) [76]. The major penalties in the freshness evaluation were determined by the presence of a sour/vinegar aroma, with a −0.59 penalty in the five-point scale, although this was noted by only about a fifth of the participants. The “dull” aspect, which was also associated with a loss of freshness (Figure 3), had a slightly higher penalty of −0.63, but the number of participants noting this was below 20%. The acid taste was noted by 22% of participants, but the impact in the freshness evaluation was only −0.10. Forty-two percent of participants noted the “dry” aspect, but its effect on the freshness evaluation was less than 0.42 points. On the positive side of the mean impact figure, the characteristic cured attributes color and aroma, as well as the aromatic notes associated with the seasoning of the cured loins (wine and garlic), had the highest positive impact on the freshness evaluation. While the smoke aroma was an attribute associated with fresher samples (Figure 3), it had a neutral impact on the freshness evaluation (Figure 4). The aspect attributes “bright”-“dull” and “moisten”-“dry” had higher impacts on the negative side than on the positive. 

### 3.2. Challenge Test with L. monocytogenes

**Figure 5:**
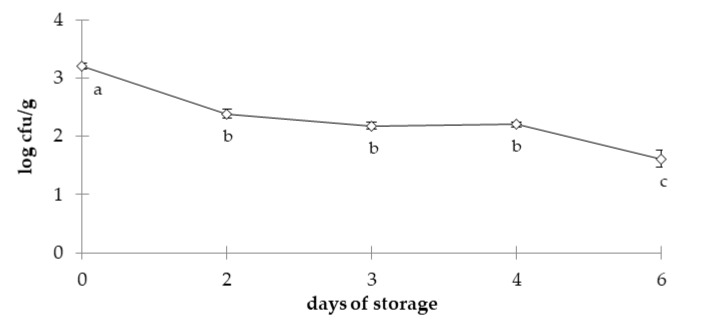
Counts of *L. monocytogenes* inoculated in cured loins stored at 6 °C for 126 days; bars indicate the standard error for each time; a, b, c means followed by different letters represent significant differences (*p* < 0.05).

Figure 5 shows the behavior of *L. monocytogenes* during the storage of sliced cured loins. The main inference from this test is that the pathogen did not grow during storage. *L. monocytogenes* has a reputation for high resistance under adverse environmental conditions [24]. In the present study, *L. monocytogenes* did not grow, probably due to the presence of nitrite. For the longest storage durations, the LAB could also contribute to pathogen inhibition Additionally, *L. monocytogenes* couts showed a slight decrease for the longest storage duration, and determined significant differences (*p* < 0.05) between 0 days (3.21 ± 0.09 log cfu/g) and all other storage durations. At 126 days, the count of the pathogen was 1.61 ± 0.25 log cfu/g, the lowest of the experiment. On average, the count of the pathogen decreased by around 1.5 log cfu/g. This behavior was probably associated with the start of the LAB growth. The first shift with a more pronounced slope—between zero and 42 days—matched the major reduction in pH observed (almost 0.6 units) (Table 3). The second shift—between 84 and 126 days—coincided with the exponential growth of LAB that dominated the microbiota of the sliced cured loins. This trend of reduced *L. monocytogenes* has been observed previously in cured products [77].

The sliced cured loins had an average aw of 0.93 and a pH of 5.8, which, according to Regulation EC 2073 [78], might support the growth of *L. monocytogenes*. Testing should be performed to guarantee that, if the product leaves the industry with *L. monocytogenes*, it does not grow [78]. The industry involved in this study follows the criteria that five 25-g samples be absent in *L. monocytogenes*. According to that legal framework, only meat products with a pH ≤ 4.4 or an aw ≤ 0.92, or products with a pH ≤ 5.0 or aw ≤ 0.94, are exempted from the need to perform challenge tests with *L. monocytogenes* if the manufacturer chooses to allow the presence of *L. monocytogenes* in the finished product. The results of the present work allow the industry to be unconcerned with this seriously concerning biological hazard. The sliced cured loins used in the present work meet all the conditions necessary to be *L. monocytogenes*-free. Besides the high level of hygiene in the industry and the qualification of suppliers, (which contribute to reducing the odds of contaminated raw materials), the hot-smoking process and presence of nitrite contribute to the elimination of potential contaminations [79]. The most severe concern for sliced meat products is accidental contamination during the slicing [24]. The industry producing sliced products only manufactures sliced meat products when they achieve very high standards of hygiene, worker training, commitment to food safety objectives, and adequate facilities and equipment, and this was the case with the company participating in this study [80]. Despite all the barriers to avoiding contamination with *L. monocytogenes*, knowing how pathogens behave in cold chains allows the industry to ponder the risks associated with sliced meat products if one accidental or unpredicted contamination occurs.

## 4. Conclusions

Sliced cured loins can be stored at 6 ± 1 °C for 105 days and maintain a consumer acceptance of more than 75%. Freshness loss is due to two concomitant mechanisms. On one hand, sliced cured loins lose intrinsic aromatic notes, particularly the smoke and cured aromas, probably due to scalping as a result of the packaging material. On the other hand, the product develops spoiled notes putatively related to the fermentative activity of LAB. A sour/vinegar aroma and acidic taste are the main sensory characteristics associated with spoilage. Any sensory trait related to rancidity or non-protein-nitrogen catabolic activity of the microbiota was detected in this study. Although lightness (L*) decreased during the storage, participants did not detect any difference in the “bright” or “dull: aspects, nor in the “moisten” or “dry” aspect.

The freshness evaluation was influenced mainly by the typical characteristics of cured products with a positive impact. The spoilage notes of a sour/vinegar aroma and acidic taste were responsible for the major penalizations in the mean evaluation of freshness.

During the period of the test, *L. monocytogenes* inoculated onto the surface of the cured loin slices did not grow. A slight reduction of about 1.5 log cfu/g was observed, and thus shelf life extension of the vacuum packaged sliced cured loins did not compromise the safety of the product.

This study had the limitation of using a convenience consumer group. As pointed out in the materials and methods section, the group was considered acceptable due to its demographic profile, consumption habits, lack of connections with the manufacturer, and small proportion of food science-related professionals.

## Figures and Tables

**Table 1:** Sampling scheme. Number of packages with approximately 10 slices each (except the challenge test, which contained five slices each).

Attribute	0 d	21 d	42 d	63 d	84 d	105 d	126 d	Total
Focus Groups								
Freshly produced (batch X)	(3 directly used; 3 frozen/thawed)							6
End of shelf life (batch Y)	9 (3 direct; 3 frozen/thawed; 3 temperature abuse)							6
Sensory—consumption freshness and CATA								
Batch 1	5 ^†^	5 ^†^	5	5	5	5	5	35
Batch 2	5	5	5	5	5	5	5	35
Microbiological and physical-chemical								
Batch 1	2	NA	1	2	1	NA	2	7
Batch 2	1	NA	2	1	2	NA	1	7
Challenge test								
Batch 1 (3 packages) ^Ψ^	2 ^ϕ^	NA	1	2	1	NA	2	3
Batch 2 (3 packages) ^Ψ^	1	NA	2	1	2	NA	1	3

^†^ Shaded in grey—samples tested in one session; not shaded—samples tested in another session. ^Ψ^ A total of 30 slices (packages × 10 slices/package) were divided into five slices/package for *L. monocytogenes* inoculation. ^ϕ^ The packages in the challenge test were prepared in our laboratory. Each package contained five slices. NA—not analyzed.

**Table 2:** Proportion of participants identifying each attribute of sliced cured loins with different storage durations.

Attribute	0 d	21 d	42 d	63 d	84 d	105 d	126 d	*p*
***Aspect***								
Moisten	0.38	0.35	0.28	0.26	0.42	0.26	0.31	0.173
Dry	0.58	0.56	0.52	0.62	0.42	0.58	0.52	0.201
Bright	0.30	0.28	0.35	0.32	0.38	0.38	0.46	0.248
Cured	0.79	0.75	0.63	0.68	0.65	0.63	0.61	0.097
Dull	0.14	0.25	0.26	0.28	0.19	0.26	0.19	0.183
***Aroma***								
Wine	0.53	0.52	0.506	0.59	0.61	0.63	0.53	0.585
Garlic	0.25	0.42	0.370	0.37	0.37	0.32	0.37	0.358
Fermented	0.03	0.05	0.074	0.07	0.11	0.07	0.07	0.519
Smoke	0.53 b	0.33 ab	0.40 ab	0.35 ab	0.27 a	0.35 ab	0.28 a	0.010
Cured	0.85 c	0.64 abc	0.70 bc	0.69 abc	0.49 ab	0.56 ab	0.46 a	0.000
Sour/vinegar	0.04	0.11	0.160	0.15	0.19	0.17	0.21	0.045 ^Ψ^
***Taste***								
Salty	0.53	0.59	0.617	0.53	0.52	0.59	0.54	0.791
Acid	0.06	0.11	0.20	0.120	0.20	0.20	0.22	0.047 ^Ψ^

a, b, c Proportions followed by different letters represent differences (*p* < 0.05). ^Ψ^ Although the *p*-value is significant, the individual differences were not significant.

**Table 3:** pH, color parameters, and microbial counts of sliced cured loins through storage duration (mean ± standard deviation of log cfu/g, *n* = 3).

Parameter	0 d	42 d	63 d	84 d	126 d	*p*
	0.93 ± 0.00	ND	ND	ND	ND	ND
pH	5.80 ± 0.01 a	5.23 ± 0.01 b	5.17 ± 0.01 c	4.99 ± 0.02 d	4.99 ± 0.04 d	<0.001
Color parameters						
L*	54.58 ± 2.28 a	52.95 ± 2.35 a	50.47 ± 0.60 a	45.05 ± 1.31 b	41.16 ± 1.57 b	<0.001
a*	22.82 ± 1.98	22.73 ± 3.03	21.47 ± 0.23	21.21 ± 1.81	20.84 ± 1.60	0.641
b*	6.45 ± 0.29	6.52 ± 0.28	6.76 ± 0.21	5.99 ± 0.43	6.47 ± 0.77	0.372
Microbial counts						
LAB	<DL d	1.79 ± 0.43 c	2.75 ± 0.20 b	7.61 ± 0.13 a	8.04 ± 0.07 a	<0.001
Mold and yeast	<DL b	<DL b	<DL b	2.69 ± 0.61 a	3.14 ± 0.79 a	<0.001

ND-not determined. DL-detection limit. a, b, c and d followed by different letters represent differences (*p* < 0.05). *Enterobacteriaceae* and *Pseudomonas* spp. were below the detection limit during all experiments (1 log cfu/g and 2 log cfu/g, respectively).

## Supplementary Materials

The following are available online at https://www.mdpi.com/2304-8158/9/5/621/s1, RAW DATA.
