# Supplementary material for: Sensory Changes and Listeria monocytogenes Behavior in Sliced Cured Pork Loins during Extended Storage
Source: Foods. 2020 May 12;9(5):621. doi: 10.3390/foods9050621 (PMC7278872; doi:10.3390/foods9050621)
Supplement: Supplementary file 1 [file foods-09-00621-s001.zip › RAW DATA CURED LOINS/RAW DATA NON-SENSORY CURED LOINS.pdf]

code	storage time	days	repetition	Count LAB	Count Mould & yeasts	Count Listeria	pH	aw	L*	a*	b*
S0A	0	0	1	0.00	0.00	3.13	5.81	0.94	52.25	20.14	6.52
S0B	0	0	2	0.00	0.00	3.20	5.80	0.94	54.69	19.30	6.14
S0C	0	0	3	0.00	0.00	3.30	5.79	0.93	56.80	23.08	6.70
S2A	2	42	1	1.92	0.00	2.52	5.24		51.61	19.59	6.67
S2B	2	42	2	1.31	0.00	2.25	5.23		51.57	19.33	6.20
S2C	2	42	3	2.15	0.00	2.38	5.23		55.66	24.70	6.69
S3A	3	63	1	2.89	0.00	2.07	5.16		50.62	21.53	6.71
S3B	3	63	2	2.51	0.00	2.29	5.18		50.98	21.21	6.99
S3C	3	63	3	2.84	0.00	2.19	5.17		49.81	21.67	6.59
S4A	4	84	1	7.53	3.27	2.19	4.98		45.56	21.68	5.70
S4B	4	84	2	7.76	2.07	2.14	5.01		43.56	21.69	5.78
S4C	4	84	3	7.54	2.72	2.29	4.98		46.02	24.82	6.49
S6A	6	126	1	8.12	3.02	1.66	4.95		39.50	22.58	5.57
S6B	6	126	2	8.03	3.99	1.83	5.00		41.35	24.52	6.69
S6C	6	126	3	7.96	2.41	1.35	5.02		42.62	21.35	7.05
