# Supplementary material for: Sensory Changes and Listeria monocytogenes Behavior in Sliced Cured Pork Loins during Extended Storage
Source: Foods. 2020 May 12;9(5):621. doi: 10.3390/foods9050621 (PMC7278872; doi:10.3390/foods9050621)
Supplement: Supplementary file 1 [file foods-09-00621-s001.zip › RAW DATA CURED LOINS/RAW DATA SENSORY CURED LOINS.pdf]

	A	B	C	D	E	F	G	H	I	J	K	L	M	N	O	P	Q	R	S	T	U	V	W	X	Y	Z	AA	AB	AC	AD	AE	AF
1	**Consumer**	Gender 0F 1M	Age (cont.)	Usual consumer 0 no 1 yes	Freshness 1 not fresh 5 very fresh	will to purchase 0 no 1 yes	**will to consume 0 no 1 yes**	**storage time**	Moisten aspect	Slime	Dry	Bright	Cured aspect	Brownish color	Dull	Darker spots	Greenish spots	Wine aroma	garlic aroma	Fermented aroma	Smoke aroma	Cured aroma	Rancid	Sulfur	Mold	Ammoniac/rotten	Butter	Sour/vinegar aroma	Salty tase	Piquant	Sweet taste	Acid taste
2	1	1	24	1	5	1	1	0	0	0	1	0	0	0	1	0	0	1	0	0	1	1	0	0	0	0	0	0	1	0	0	0
3	2	0	23	1	5	1	1	0	1	0	0	0	1	1	0	0	0	0	0	0	1	1	0	0	0	0	0	0	0	0	0	0
4	3	1	53	1	5	1	0	0	1	0	0	1	1	0	0	1	0	1	1	0	1	1	0	0	0	0	0	0	1	0	0	0
5	4	0	52	1	5	1	0	0	1	0	0	1	1	0	0	0	0	1	1	0	1	0	0	0	0	0	0	0	1	0	0	0
6	5	0	23	1	5	1	1	0	0	0	1	1	1	0	0	0	0	1	0	0	0	1	0	0	0	0	0	0	1	0	0	0
7	6	0	23	1	4	1	1	0	0	0	1	0	0	1	0	0	0	0	1	0	0	0	0	0	0	0	0	0	1	0	0	0
8	7	1	55	1	4	1	1	0	0	0	1	0	1	0	0	0	0	0	0	0	0	1	0	0	0	0	0	0	1	0	0	0
9	8	0	54	1	5	1	1	0	0	0	1	0	1	0	0	0	0	1	1	0	1	1	0	0	0	0	0	0	1	0	0	0
10	9	1	21	1	4	1	1	0	0	0	1	0	1	1	0	0	0	1	1	0	1	1	0	0	0	0	0	0	0	0	1	0
11	10	1	55	1	5	1	1	0	0	0	1	1	1	0	0	0	0	0	0	0	0	1	0	0	0	0	0	0	1	0	0	0
12	11	0	23	0	5	1	1	0	1	0	0	0	1	0	0	0	0	0	0	0	1	1	0	0	0	0	0	0	1	0	0	0
13	12	0	52	1	4	1	1	0	1	0	0	0	1	0	0	0	0	0	0	0	0	1	0	0	0	0	0	0	1	0	0	0
14	13	1	50	0	5	1	1	0	0	0	1	0	1	0	0	0	0	0	0	0	1	0	0	0	0	0	0	0	1	0	0	0
15	14	1	85	1	5	1	1	0	1	0	0	0	1	0	0	0	0	0	0	0	1	1	0	0	0	0	0	0	1	0	0	0
16	15	1	60	0	4	1	1	0	0	0	1	0	1	0	1	0	0	0	0	0	1	1	0	0	0	0	0	0	1	0	0	0
17	16	0	58	1	4	1	1	0	0	0	1	0	1	0	0	0	0	1	0	0	0	1	0	0	0	0	0	0	0	1	0	0
18	17	1	30	1	3	0	0	0	1	0	0	0	0	0	1	0	0	1	0	0	1	0	0	0	0	0	0	0	1	0	0	0
19	18	0	59	1	4	1	1	0	0	1	0	1	0	0	0	0	0	0	0	0	1	1	0	0	0	0	0	0	1	0	0	0
20	19	0	23	1	4	1	1	0	0	0	1	0	1	0	1	0	0	1	1	0	0	1	0	0	0	0	0	1	1	0	0	1
21	20	0	22	0	5	1	1	0	0	0	1	0	1	0	0	0	0	1	0	0	0	1	0	0	0	0	0	0	0	0	0	0

	A	B	C	D	E	F	G	H	I	J	K	L	M	N	O	P	Q	R	S	T	U	V	W	X	Y	Z	AA	AB	AC	AD	AE	AF
1	**Consumer**	Gender 0F 1M	Age (cont.)	Usual consumer 0 no 1 yes	Freshness 1 not fresh 5 very fresh	will to purchase 0 no 1 yes	**will to consume 0 no 1 yes**	**storage time**	Moisten aspect	Slime	Dry	Bright	Cured aspect	Brownish color	Dull	Darker spots	Greenish spots	Wine aroma	garlic aroma	Fermented aroma	Smoke aroma	Cured aroma	Rancid	Sulfur	Mold	Ammoniac/rotten	Butter	Sour/vinegar aroma	Salty tase	Piquant	Sweet taste	Acid taste
22	21	0	23	1	5	1	1	0	0	0	1	0	1	0	0	0	0	1	0	0	0	1	0	0	0	0	0	0	0	0	0	0
23	22	0	28	1	5	1	1	0	0	0	1	0	1	0	0	0	0	1	0	0	1	1	0	0	0	0	0	0	0	0	0	0
24	23	1	23	1	4	1	1	0	0	0	1	1	0	0	0	1	0	1	0	0	0	1	0	0	0	0	0	0	1	0	0	0
25	24	1	39	1	3	1	1	0	1	1	0	1	1	1	0	0	0	1	0	0	0	1	0	0	0	0	0	0	0	0	0	0
26	25	0	26	1	4	1	1	0	1	0	0	0	1	0	0	0	0	0	0	0	1	1	0	0	0	0	0	0	0	1	0	0
27	26	0	27	1	4	1	1	0	0	0	1	0	1	0	0	0	0	0	1	0	0	1	0	0	0	0	0	0	0	1	0	0
28	27	0	36	1	3	1	1	0	0	0	1	1	1	0	0	0	0	0	0	0	1	1	0	0	0	0	0	0	0	1	0	0
29	28	1	49	1	5	1	1	0	0	0	1	0	1	0	0	0	0	1	0	0	1	1	0	0	0	0	1	0	0	1	0	0
30	29	1	23	0	3	1	1	0	0	0	1	0	0	1	0	0	0	1	0	1	0	0	0	0	0	0	0	0	0	0	1	1
31	30	0	46	1	4	1	1	0	1	0	0	0	1	0	0	0	0	0	0	0	0	1	0	0	0	0	0	0	0	1	0	0
32	31	1	47	1	4	1	1	0	1	0	0	1	1	0	0	0	0	0	0	0	1	1	0	0	0	0	0	0	0	1	0	0
33	32	0	53	1	4	1	1	0	0	0	1	0	1	0	0	0	0	1	0	0	1	1	0	0	0	0	0	0	0	0	0	0
34	33	0	48	1	4	1	1	0	1	0	0	0	1	0	0	0	0	0	0	0	0	1	0	0	0	0	0	0	0	1	0	0
35	34	1	52	1	4	1	1	0	1	0	0	1	0	0	0	0	0	1	0	0	0	1	0	0	0	0	0	0	1	0	0	0
36	35	0	23	1	3	0	1	0	0	0	1	0	0	0	1	0	0	1	1	0	0	0	0	0	0	0	0	0	1	0	0	0
37	36	0	44	0	3	1	1	0	1	0	0	0	1	0	0	0	0	0	0	0	1	1	0	0	0	0	0	0	0	0	1	0
38	37	0	26	1	4	1	1	0	1	0	0	1	1	0	0	0	0	1	1	0	1	1	0	0	0	0	0	0	1	0	1	0
39	38	0	26	1	5	1	1	0	0	0	1	0	0	0	1	0	0	1	0	0	1	1	0	0	0	0	0	0	1	0	0	0
40	39	0	44	1	5	1	1	0	1	0	0	0	1	1	0	0	0	0	0	0	1	1	0	0	0	0	0	0	0	0	0	0
41	40	0	21	1	5	1	0	0	1	0	0	1	1	0	0	1	0	1	1	0	1	1	0	0	0	0	0	0	1	0	0	0

	A	B	C	D	E	F	G	H	I	J	K	L	M	N	O	P	Q	R	S	T	U	V	W	X	Y	Z	AA	AB	AC	AD	AE	AF
1	**Consumer**	Gender 0F 1M	Age (cont.)	Usual consumer 0 no 1 yes	Freshness 1 not fresh 5 very fresh	will to purchase 0 no 1 yes	**will to consume 0 no 1 yes**	**storage time**	Moisten aspect	Slime	Dry	Bright	Cured aspect	Brownish color	Dull	Darker spots	Greenish spots	Wine aroma	garlic aroma	Fermented aroma	Smoke aroma	Cured aroma	Rancid	Sulfur	Mold	Ammoniac/rotten	Butter	Sour/vinegar aroma	Salty tase	Piquant	Sweet taste	Acid taste
42	41	0	28	1	5	1	0	0	1	0	0	1	1	0	0	0	0	1	1	0	1	0	0	0	0	0	0	0	1	0	0	0
43	42	0	24	1	5	1	1	0	0	0	1	1	1	0	0	0	0	1	0	0	0	1	0	0	0	0	0	0	1	0	0	0
44	43	0	23	1	4	1	1	0	0	0	1	0	0	1	0	0	0	0	1	0	0	0	0	0	0	0	0	0	1	0	0	0
45	44	1	32	1	4	1	1	0	0	0	1	0	1	0	0	0	0	0	0	0	0	1	0	0	0	0	0	0	1	0	0	0
46	45	1	55	1	5	1	1	0	0	0	1	0	1	0	0	0	0	1	1	0	1	1	0	0	0	0	0	0	1	0	0	0
47	46	1	54	1	4	1	1	0	0	0	1	0	1	1	0	0	0	1	1	0	1	1	0	0	0	0	0	0	0	0	1	0
48	47	0	57	0	5	1	1	0	0	0	1	1	1	0	0	0	0	0	0	0	0	1	0	0	0	0	0	0	1	0	0	0
49	48	1	42	1	5	1	1	0	1	0	0	0	1	0	0	0	0	0	0	0	1	1	0	0	0	0	0	0	1	0	0	0
50	49	0	26	1	4	1	1	0	1	0	0	0	1	0	0	0	0	0	0	0	0	1	0	0	0	0	0	0	1	0	0	0
51	50	0	56	1	5	1	1	0	0	0	1	0	1	0	0	0	0	0	0	0	1	0	0	0	0	0	0	0	1	0	0	0
52	51	1	29	1	5	1	1	0	1	0	0	0	1	0	0	0	0	0	0	0	1	1	0	0	0	0	0	0	1	0	0	0
53	52	0	26	1	4	1	1	0	0	0	1	0	1	0	1	0	0	0	0	0	1	1	0	0	0	0	0	0	1	0	0	0
54	53	0	26	0	4	1	1	0	0	0	1	0	1	0	0	0	0	1	0	0	0	1	0	0	0	0	0	0	0	1	0	0
55	54	0	60	1	3	0	0	0	1	0	0	0	0	0	1	0	0	1	0	0	1	0	0	0	0	0	0	0	1	0	0	0
56	55	0	50	1	4	1	1	0	0	1	0	1	0	0	0	0	0	0	0	0	1	1	0	0	0	0	0	0	1	0	0	0
57	56	0	54	1	4	1	1	0	0	0	1	0	1	0	1	0	0	1	1	0	0	1	0	0	0	0	0	1	1	0	0	1
58	57	1	38	1	5	1	1	0	0	0	1	0	1	0	0	0	0	1	0	0	0	1	0	0	0	0	0	0	0	0	0	0
59	58	0	24	1	5	1	1	0	0	0	1	0	1	0	0	0	0	1	0	0	0	1	0	0	0	0	0	0	0	0	0	0
60	59	0	23	1	5	1	1	0	0	0	1	0	1	0	0	0	0	1	0	0	1	1	0	0	0	0	0	0	0	0	0	0
61	60	1	22	1	4	1	1	0	0	0	1	1	0	0	0	1	0	1	0	0	0	1	0	0	0	0	0	0	1	0	0	0

	A	B	C	D	E	F	G	H	I	J	K	L	M	N	O	P	Q	R	S	T	U	V	W	X	Y	Z	AA	AB	AC	AD	AE	AF
1	**Consumer**	Gender 0F 1M	Age (cont.)	Usual consumer 0 no 1 yes	Freshness 1 not fresh 5 very fresh	will to purchase 0 no 1 yes	**will to consume 0 no 1 yes**	**storage time**	Moisten aspect	Slime	Dry	Bright	Cured aspect	Brownish color	Dull	Darker spots	Greenish spots	Wine aroma	garlic aroma	Fermented aroma	Smoke aroma	Cured aroma	Rancid	Sulfur	Mold	Ammoniac/rotten	Butter	Sour/vinegar aroma	Salty tase	Piquant	Sweet taste	Acid taste
62	61	1	35	1	3	1	1	0	1	1	0	1	1	1	0	0	0	1	0	0	0	1	0	0	0	0	0	0	0	0	0	0
63	62	1	58	1	4	1	1	0	1	0	0	0	1	0	0	0	0	0	0	0	1	1	0	0	0	0	0	0	0	1	0	0
64	63	0	26	1	4	1	1	0	0	0	1	0	1	0	0	0	0	0	1	0	0	1	0	0	0	0	0	0	0	1	0	0
65	64	0	56	1	3	1	1	0	0	0	1	1	1	0	0	0	0	0	0	0	1	1	0	0	0	0	0	0	0	1	0	0
66	65	0	33	1	5	1	1	0	0	0	1	0	1	0	0	0	0	1	0	0	1	1	0	0	0	0	1	0	0	1	0	0
67	66	0	54	1	3	1	1	0	0	0	1	0	0	1	0	0	0	1	0	1	0	0	0	0	0	0	0	0	0	0	1	1
68	67	1	25	1	4	1	1	0	1	0	0	0	1	0	0	0	0	0	0	0	0	1	0	0	0	0	0	0	0	1	0	0
69	68	1	25	1	4	1	1	0	1	0	0	1	1	0	0	0	0	0	0	0	1	1	0	0	0	0	0	0	0	1	0	0
70	69	1	29	1	4	1	1	0	0	0	1	0	1	0	0	0	0	1	0	0	1	1	0	0	0	0	0	0	0	0	0	0
71	70	1	56	1	4	1	1	0	1	0	0	0	1	0	0	0	0	0	0	0	0	1	0	0	0	0	0	0	0	1	0	0
72	71	1	50	1	4	1	1	0	1	0	0	1	0	0	0	0	0	1	0	0	0	1	0	0	0	0	0	0	1	0	0	0
73	72	0	57	1	3	0	1	0	0	0	1	0	0	0	1	0	0	1	1	0	0	0	0	0	0	0	0	0	1	0	0	0
74	73	1	22	1	3	1	1	0	1	0	0	0	1	0	0	0	0	0	0	0	1	1	0	0	0	0	0	0	0	0	1	0
75	74	1	55	1	4	1	1	0	1	0	0	1	1	0	0	0	0	1	1	0	1	1	0	0	0	0	0	0	1	0	1	0
76	75	0	26	1	4	1	1	0	0	0	1	0	1	0	0	0	0	0	1	0	0	1	0	0	0	0	0	0	0	1	0	0
77	76	0	53	1	3	1	1	0	0	0	1	1	1	0	0	0	0	0	0	0	1	1	0	0	0	0	0	0	0	1	0	0
78	77	1	53	1	5	1	1	0	0	0	1	0	1	0	0	0	0	1	0	0	1	1	0	0	0	0	1	0	0	1	0	0
79	78	1	58	1	4	1	1	0	0	1	0	1	0	0	0	0	0	0	0	0	1	1	0	0	0	0	0	0	1	0	0	0
80	79	0	60	1	4	1	1	0	0	0	1	0	1	0	1	0	0	1	1	0	0	1	0	0	0	0	0	1	1	0	0	1
81	80	1	26	1	5	1	1	0	0	0	1	0	1	0	0	0	0	1	0	0	0	1	0	0	0	0	0	0	0	0	0	0

	A	B	C	D	E	F	G	H	I	J	K	L	M	N	O	P	Q	R	S	T	U	V	W	X	Y	Z	AA	AB	AC	AD	AE	AF
1	**Consumer**	Gender 0F 1M	Age (cont.)	Usual consumer 0 no 1 yes	Freshness 1 not fresh 5 very fresh	will to purchase 0 no 1 yes	**will to consume 0 no 1 yes**	**storage time**	Moisten aspect	Slime	Dry	Bright	Cured aspect	Brownish color	Dull	Darker spots	Greenish spots	Wine aroma	garlic aroma	Fermented aroma	Smoke aroma	Cured aroma	Rancid	Sulfur	Mold	Ammoniac/rotten	Butter	Sour/vinegar aroma	Salty tase	Piquant	Sweet taste	Acid taste
82	81	0	27	1	4	1	1	0	1	0	0	0	1	0	0	0	0	0	0	0	0	1	0	0	0	0	0	0	1	0	0	0
83	1	1	24	1	2	1	**1**	21	**0**	0	1	0	0	1	1	0	0	0	0	0	1	1	0	0	0	0	0	1	1	0	1	1
84	2	0	23	1	4	1	1	21	0	0	1	0	0	0	1	0	0	1	0	0	0	0	0	0	0	0	0	0	1	0	0	0
85	3	1	53	1	4	1	1	21	1	0	0	0	1	0	0	0	0	1	0	0	0	0	0	0	0	0	0	0	1	0	0	0
86	4	0	52	1	3	1	1	21	1	0	0	1	1	0	0	0	0	1	1	0	1	0	0	0	0	0	0	0	1	0	0	0
87	5	0	23	1	5	1	1	21	0	0	1	1	1	0	0	0	0	1	1	0	0	1	0	0	0	0	0	0	0	0	1	0
88	6	0	23	1	4	1	1	21	0	0	1	0	1	0	0	0	0	1	1	0	0	1	0	0	0	0	0	0	1	0	0	0
89	7	1	55	1	2	0	0	21	0	0	1	0	1	0	1	0	0	1	1	0	1	1	0	0	0	0	0	1	1	0	0	1
90	8	0	54	1	5	1	1	21	0	0	1	1	1	0	0	0	0	1	0	0	1	1	0	0	0	0	0	0	1	0	0	0
91	9	1	21	1	4	1	1	21	0	1	0	0	1	0	0	0	0	0	0	0	1	1	0	0	0	0	0	0	1	0	0	0
92	10	1	55	1	4	1	1	21	0	0	1	0	1	0	1	0	0	1	1	0	1	1	0	0	0	0	0	1	1	0	0	1
93	11	0	23	0	4	1	1	21	1	1	0	1	1	0	0	0	0	0	1	0	1	1	0	0	0	0	0	0	1	0	0	0
94	12	0	52	1	3	1	1	21	0	0	1	0	1	0	0	0	0	0	0	0	1	1	0	0	0	0	0	0	1	0	0	0
95	13	1	50	0	4	1	1	21	0	0	1	0	1	0	0	0	0	1	0	0	1	1	0	0	0	0	0	0	0	0	0	0
96	14	1	85	1	3	1	1	21	1	0	0	0	0	0	1	0	0	0	0	0	0	1	0	0	0	0	0	0	1	0	0	0
97	15	1	60	0	3	1	0	21	0	0	1	0	1	0	0	0	0	0	0	0	0	0	0	0	0	0	0	0	1	0	0	0
98	16	0	58	1	5	1	1	21	0	0	1	0	1	0	0	0	0	0	1	0	0	1	0	0	0	0	0	0	1	0	0	0
99	17	1	30	1	5	1	1	21	0	0	1	0	1	0	0	0	0	1	1	0	0	1	0	0	0	0	0	0	1	0	0	0
100	18	0	59	1	3	0	1	21	0	0	1	0	1	0	0	0	0	0	0	0	0	0	0	0	1	0	0	0	1	0	0	0
101	19	0	23	1	3	0	0	21	0	0	1	0	1	1	1	0	0	0	0	0	1	1	0	0	0	0	0	1	1	0	0	0

	A	B	C	D	E	F	G	H	I	J	K	L	M	N	O	P	Q	R	S	T	U	V	W	X	Y	Z	AA	AB	AC	AD	AE	AF
1	**Consumer**	Gender 0F 1M	Age (cont.)	Usual consumer 0 no 1 yes	Freshness 1 not fresh 5 very fresh	will to purchase 0 no 1 yes	**will to consume 0 no 1 yes**	**storage time**	Moisten aspect	Slime	Dry	Bright	Cured aspect	Brownish color	Dull	Darker spots	Greenish spots	Wine aroma	garlic aroma	Fermented aroma	Smoke aroma	Cured aroma	Rancid	Sulfur	Mold	Ammoniac/rotten	Butter	Sour/vinegar aroma	Salty tase	Piquant	Sweet taste	Acid taste
102	20	0	22	0	5	1	1	21	0	0	1	0	0	0	1	0	0	1	0	0	0	1	0	0	0	0	0	1	1	0	0	0
103	21	0	23	1	5	1	1	21	0	0	1	0	1	0	0	0	0	1	1	0	0	1	0	0	0	0	0	0	1	0	0	0
104	22	0	28	1	4	1	1	21	0	0	1	1	1	0	0	0	0	0	1	0	1	0	0	0	0	0	0	0	1	0	0	0
105	23	1	23	1	4	1	1	21	1	0	0	0	1	0	0	0	0	1	0	0	0	0	0	0	0	0	0	0	1	0	0	0
106	24	1	39	1	5	1	1	21	0	0	1	0	1	0	0	0	0	1	0	0	0	0	0	0	0	0	0	0	1	0	0	0
107	25	0	26	1	3	1	1	21	0	0	1	0	1	0	0	0	0	0	0	0	0	1	0	0	0	0	0	0	0	0	0	0
108	26	0	27	1	4	1	1	21	0	0	1	0	1	0	0	0	0	0	0	0	0	1	0	0	0	0	0	0	0	0	0	0
109	27	0	36	1	4	1	1	21	0	1	0	1	0	0	0	0	0	1	0	0	0	1	0	0	0	0	0	0	1	0	0	0
110	28	1	49	1	1	0	0	21	0	0	1	1	1	0	0	0	0	1	1	0	1	1	0	0	0	0	0	0	1	0	0	0
111	29	1	23	0	5	1	1	21	1	0	0	0	1	0	0	0	0	1	0	0	1	1	0	0	0	0	0	0	1	0	0	0
112	30	0	46	1	4	1	1	21	0	0	1	1	0	0	0	0	0	1	1	0	0	0	0	0	0	0	0	0	0	0	0	0
113	31	1	47	1	5	1	1	21	0	1	0	0	1	0	0	0	0	0	1	0	0	0	0	0	0	0	0	0	0	0	0	0
114	32	0	53	1	4	1	1	21	0	0	1	0	1	0	0	0	0	0	0	0	1	0	0	0	0	0	0	0	0	0	0	0
115	33	0	48	1	5	1	1	21	1	0	0	0	1	0	0	0	0	1	0	0	0	0	0	0	0	0	0	0	1	0	0	0
116	34	1	52	1	3	1	1	21	0	0	1	0	1	0	0	0	0	1	0	0	0	1	0	0	0	0	0	0	0	0	0	0
117	35	0	23	1	4	1	1	21	0	0	1	0	1	0	0	0	0	0	0	0	1	0	0	0	0	0	0	0	1	0	0	0
118	36	0	44	0	4	1	1	21	0	0	1	0	1	0	0	0	0	1	0	0	0	1	0	0	0	0	0	0	0	0	0	0
119	37	0	26	1	3	1	1	21	1	0	0	0	0	0	1	0	0	1	0	0	0	0	0	0	0	0	0	0	0	1	0	0
120	38	0	26	1	4	1	1	21	1	0	0	0	1	0	0	0	0	0	0	0	0	1	0	0	0	0	0	0	1	0	0	0
121	39	0	44	1	3	1	1	21	0	0	1	0	1	0	1	0	0	1	0	0	0	1	0	0	0	0	0	0	0	0	1	0

	A	B	C	D	E	F	G	H	I	J	K	L	M	N	O	P	Q	R	S	T	U	V	W	X	Y	Z	AA	AB	AC	AD	AE	AF
1	**Consumer**	Gender 0F 1M	Age (cont.)	Usual consumer 0 no 1 yes	Freshness 1 not fresh 5 very fresh	will to purchase 0 no 1 yes	**will to consume 0 no 1 yes**	**storage time**	Moisten aspect	Slime	Dry	Bright	Cured aspect	Brownish color	Dull	Darker spots	Greenish spots	Wine aroma	garlic aroma	Fermented aroma	Smoke aroma	Cured aroma	Rancid	Sulfur	Mold	Ammoniac/rotten	Butter	Sour/vinegar aroma	Salty tase	Piquant	Sweet taste	Acid taste
122	40	0	21	1	3	1	1	21	1	0	0	1	0	0	0	0	0	0	0	0	0	0	0	0	0	0	0	0	1	0	0	1
123	41	0	28	1	4	1	1	21	1	0	0	0	1	1	0	0	0	1	0	0	0	1	0	0	0	0	0	0	0	0	0	0
124	42	0	24	1	3	1	1	21	1	0	0	1	0	0	0	0	0	0	0	0	0	1	0	0	0	0	0	0	0	0	0	0
125	43	0	23	1	3	1	1	21	1	0	0	0	1	0	0	0	0	1	1	0	0	0	0	0	0	0	0	0	1	0	0	0
126	44	1	32	1	3	1	1	21	1	0	0	1	0	0	0	0	0	0	0	0	0	1	0	0	0	0	0	0	0	0	0	0
127	45	1	55	1	4	1	1	21	0	0	1	0	1	0	0	0	0	1	1	1	1	1	0	0	0	0	0	0	1	1	1	1
128	46	1	54	1	5	0	1	21	1	0	0	0	1	0	0	0	0	0	0	0	0	1	0	0	0	0	0	0	0	0	1	0
129	47	0	57	0	4	1	1	21	1	0	0	1	0	0	0	0	0	0	0	0	0	1	0	0	0	0	0	0	1	0	0	0
130	48	1	42	1	4	1	1	21	0	0	0	1	0	0	0	1	0	1	1	0	0	1	0	0	0	0	0	0	1	0	1	0
131	49	0	26	1	5	1	1	21	1	0	0	0	1	0	0	0	0	1	1	0	0	1	0	0	0	0	0	0	0	0	0	0
132	50	0	56	1	5	1	1	21	1	0	1	1	1	0	0	0	0	0	1	0	1	1	0	0	0	0	0	0	0	1	0	0
133	51	1	29	1	4	0	1	21	0	1	0	0	1	0	0	0	0	0	1	0	0	1	0	0	0	0	0	0	0	0	0	1
134	52	0	26	1	4	1	1	21	0	0	1	0	1	0	1	0	0	0	0	0	1	0	0	0	0	0	0	0	1	0	0	0
135	53	0	26	0	4	1	1	21	1	0	0	1	1	0	0	0	0	0	1	1	0	1	0	0	0	0	0	0	1	0	0	1
136	54	0	60	1	4	1	1	21	0	1	0	1	1	0	0	0	0	1	1	0	0	0	0	0	0	0	0	0	0	0	0	0
137	55	0	50	1	3	1	1	21	0	0	1	0	1	0	1	0	0	1	1	0	1	1	0	0	0	0	0	0	1	0	0	0
138	56	0	54	1	1	0	0	21	0	0	1	0	0	1	1	0	0	1	1	1	0	0	0	0	0	0	0	1	1	0	0	0
139	57	1	38	1	2	0	1	21	1	0	0	1	0	0	0	0	0	1	0	0	0	1	0	0	0	0	0	1	0	0	0	0
140	58	0	24	1	3	1	1	21	1	0	0	0	1	0	0	0	0	1	1	0	1	1	0	0	0	0	0	0	0	0	1	0
141	59	0	23	1	3	1	1	21	1	0	0	0	1	0	0	0	0	0	1	0	0	0	0	0	0	0	1	0	0	0	0	0

	A	B	C	D	E	F	G	H	I	J	K	L	M	N	O	P	Q	R	S	T	U	V	W	X	Y	Z	AA	AB	AC	AD	AE	AF
1	**Consumer**	Gender 0F 1M	Age (cont.)	Usual consumer 0 no 1 yes	Freshness 1 not fresh 5 very fresh	will to purchase 0 no 1 yes	**will to consume 0 no 1 yes**	**storage time**	Moisten aspect	Slime	Dry	Bright	Cured aspect	Brownish color	Dull	Darker spots	Greenish spots	Wine aroma	garlic aroma	Fermented aroma	Smoke aroma	Cured aroma	Rancid	Sulfur	Mold	Ammoniac/rotten	Butter	Sour/vinegar aroma	Salty tase	Piquant	Sweet taste	Acid taste
142	60	1	22	1	4	1	1	21	0	0	1	1	1	0	0	0	0	1	1	0	0	0	0	0	0	0	0	0	1	0	1	1
143	61	1	35	1	3	1	1	21	0	0	1	0	1	0	1	0	0	1	1	0	0	1	0	0	0	0	0	0	0	0	1	0
144	62	1	58	1	4	1	1	21	1	0	0	1	1	1	0	0	0	1	1	0	0	0	0	0	0	0	0	0	0	0	0	0
145	63	0	26	1	3	1	1	21	0	1	1	0	1	0	1	0	0	0	0	1	0	1	0	0	0	0	0	1	0	0	0	1
146	64	0	56	1	5	1	1	21	0	0	1	0	1	0	1	0	0	1	1	0	1	1	0	0	0	0	0	0	1	0	0	0
147	65	0	33	1	4	1	1	21	0	1	0	0	0	1	0	0	0	0	1	0	0	1	0	0	0	0	0	0	0	0	0	0
148	66	0	54	1	5	1	1	21	0	0	1	0	1	0	1	0	0	1	1	0	0	1	0	0	0	0	0	0	0	0	0	0
149	67	1	25	1	3	0	1	21	0	0	1	0	1	0	1	0	0	0	0	0	0	1	0	0	0	0	0	0	0	0	1	0
150	68	1	25	1	5	1	1	21	0	0	0	0	1	0	0	0	0	1	1	0	1	0	0	0	0	0	0	0	0	0	0	0
151	69	1	29	1	4	1	1	21	0	0	1	0	1	0	0	0	0	0	0	0	1	1	0	0	0	0	0	0	0	0	1	0
152	70	1	56	1	5	1	1	21	0	0	1	0	1	0	0	0	0	0	0	0	0	1	0	0	0	0	0	0	1	0	0	0
153	71	1	50	1	5	1	1	21	0	0	1	0	0	0	0	1	0	0	0	0	0	0	0	0	0	0	0	0	0	0	1	0
154	72	0	57	1	3	1	1	21	1	0	0	1	1	0	0	0	0	1	1	0	1	1	0	0	0	0	0	0	1	0	0	0
155	73	1	22	1	4	1	1	21	1	0	0	0	1	0	0	0	0	0	0	0	0	1	1	0	0	0	0	0	0	0	0	0
156	74	1	55	1	5	1	1	21	0	0	1	0	1	0	1	0	0	0	0	0	1	1	0	0	0	0	0	0	0	0	1	0
157	75	0	26	1	4	1	1	21	1	0	0	1	0	0	0	0	0	0	0	0	1	0	0	0	0	0	0	0	1	0	0	0
158	76	0	53	1	4	1	1	21	0	0	1	0	1	0	1	1	0	1	1	0	1	1	0	0	0	0	0	1	1	0	0	0
159	77	1	53	1	4	1	1	21	1	0	0	1	0	0	0	0	0	1	0	0	0	0	0	0	0	0	0	0	1	0	0	0
160	78	1	58	1	4	0	1	21	0	0	1	0	1	0	0	0	0	0	0	0	0	0	0	0	0	0	0	0	1	0	0	0
161	79	0	60	1	5	1	1	21	0	0	1	0	1	0	0	0	0	0	0	0	0	1	0	0	0	0	0	0	1	0	0	0

	A	B	C	D	E	F	G	H	I	J	K	L	M	N	O	P	Q	R	S	T	U	V	W	X	Y	Z	AA	AB	AC	AD	AE	AF
1	**Consumer**	Gender 0F 1M	Age (cont.)	Usual consumer 0 no 1 yes	Freshness 1 not fresh 5 very fresh	will to purchase 0 no 1 yes	**will to consume 0 no 1 yes**	**storage time**	Moisten aspect	Slime	Dry	Bright	Cured aspect	Brownish color	Dull	Darker spots	Greenish spots	Wine aroma	garlic aroma	Fermented aroma	Smoke aroma	Cured aroma	Rancid	Sulfur	Mold	Ammoniac/rotten	Butter	Sour/vinegar aroma	Salty tase	Piquant	Sweet taste	Acid taste
162	80	1	26	1	5	1	1	21	1	0	0	1	0	0	0	0	0	0	0	0	0	0	0	0	0	0	0	0	1	0	0	0
163	81	0	27	1	4	1	1	21	0	1	0	0	0	0	1	0	0	0	0	0	0	0	0	0	0	0	0	0	1	0	0	0
164	1	1	24	1	3	1	1	42	0	0	1	0	0	1	1	0	0	1	0	1	0	1	0	0	0	0	0	1	1	0	0	1
165	2	0	23	1	3	0	1	42	0	0	1	0	0	0	1	0	0	1	0	0	0	0	0	0	0	0	0	1	0	0	0	1
166	3	1	53	1	2	0	0	42	1	0	0	0	1	0	0	0	0	0	0	0	0	1	0	0	0	0	0	0	0	0	0	1
167	4	0	52	1	4	1	1	42	1	0	0	1	1	0	0	0	0	0	0	0	1	0	0	0	0	0	0	0	1	0	0	0
168	5	0	23	1	4	1	1	42	0	0	1	0	1	0	0	0	0	0	0	0	0	1	0	0	0	0	0	0	1	0	0	0
169	6	0	23	1	3	1	1	42	0	0	1	0	1	0	0	0	0	1	1	0	0	1	0	0	0	0	0	0	1	0	0	0
170	7	1	55	1	4	1	1	42	1	0	0	1	1	0	0	0	0	1	1	0	1	1	0	0	0	0	0	0	1	0	0	0
171	8	0	54	1	4	1	1	42	0	0	1	0	0	1	1	0	0	1	0	0	1	1	0	0	0	0	0	0	1	0	0	0
172	9	1	21	1	3	0	0	42	0	1	1	1	0	0	0	1	0	1	1	0	0	1	0	0	0	0	0	0	1	0	0	0
173	10	1	55	1	3	1	1	42	0	0	1	0	1	0	1	0	0	0	0	0	1	1	0	0	0	0	0	0	0	0	0	1
174	11	0	23	0	3	1	0	42	0	0	1	0	1	0	1	0	0	1	1	0	1	1	0	0	0	0	0	1	1	0	0	1
175	12	0	52	1	5	1	1	42	0	0	0	1	1	0	0	0	0	1	1	0	1	1	0	0	0	0	0	0	1	0	0	0
176	13	1	50	0	3	1	1	42	0	0	1	0	1	0	0	0	0	1	0	0	1	1	0	0	0	0	0	0	1	0	0	0
177	14	1	85	1	3	1	1	42	0	0	1	0	1	0	1	0	0	1	0	0	1	1	0	0	0	0	0	0	1	0	0	0
178	15	1	60	0	3	1	1	42	0	0	1	0	1	0	0	0	0	0	0	0	0	1	0	0	0	0	0	0	1	0	0	0
179	16	0	58	1	3	0	1	42	0	0	1	0	1	0	0	0	0	1	1	0	0	1	0	0	0	0	0	0	0	0	0	0
180	17	1	30	1	5	1	1	42	0	0	1	0	1	0	0	0	0	0	1	0	0	1	0	0	0	0	0	0	1	0	0	0
181	18	0	59	1	5	1	1	42	0	0	1	0	1	0	0	0	0	1	1	1	0	1	0	0	0	0	0	0	1	0	0	0

	A	B	C	D	E	F	G	H	I	J	K	L	M	N	O	P	Q	R	S	T	U	V	W	X	Y	Z	AA	AB	AC	AD	AE	AF
1	**Consumer**	Gender 0F 1M	Age (cont.)	Usual consumer 0 no 1 yes	Freshness 1 not fresh 5 very fresh	will to purchase 0 no 1 yes	**will to consume 0 no 1 yes**	**storage time**	Moisten aspect	Slime	Dry	Bright	Cured aspect	Brownish color	Dull	Darker spots	Greenish spots	Wine aroma	garlic aroma	Fermented aroma	Smoke aroma	Cured aroma	Rancid	Sulfur	Mold	Ammoniac/rotten	Butter	Sour/vinegar aroma	Salty tase	Piquant	Sweet taste	Acid taste
182	19	0	23	1	2	0	0	42	0	1	0	0	0	0	1	0	0	0	0	0	0	0	0	0	0	0	0	1	0	0	0	1
183	20	0	22	0	1	0	0	42	0	0	1	0	0	1	1	1	0	0	0	0	1	1	1	0	1	0	0	1	1	0	0	0
184	21	0	23	1	3	0	1	42	0	0	1	0	0	0	1	0	0	0	1	0	0	1	0	0	0	0	0	0	1	0	0	0
185	22	0	28	1	4	1	1	42	1	0	0	0	0	0	1	0	0	1	0	0	0	0	0	0	0	0	0	0	1	0	0	0
186	23	1	23	1	4	1	1	42	0	1	0	0	1	0	0	0	0	0	0	0	0	1	0	0	0	0	0	0	1	0	0	0
187	24	1	39	1	4	1	1	42	1	0	0	1	1	0	0	1	0	1	1	0	1	1	0	0	0	0	0	0	0	0	0	0
188	25	0	26	1	4	1	1	42	1	0	0	0	1	0	0	0	0	1	1	0	0	1	0	0	0	0	0	0	1	0	0	1
189	26	0	27	1	4	1	1	42	0	1	1	1	1	1	0	0	0	1	1	0	1	1	0	0	0	0	0	0	1	0	1	0
190	27	0	36	1	5	1	1	42	1	0	0	1	1	0	0	0	0	0	1	0	1	1	0	0	0	0	0	0	1	0	0	0
191	28	1	49	1	4	1	1	42	1	0	0	1	1	1	0	0	0	1	1	0	0	0	0	0	0	0	0	0	0	0	0	0
192	29	1	23	0	4	1	1	42	0	1	1	0	0	0	1	1	0	0	0	0	0	1	0	0	0	0	0	0	0	0	0	1
193	30	0	46	1	4	1	1	42	0	0	1	0	1	0	1	0	0	1	1	0	1	1	0	0	0	0	0	1	1	0	0	0
194	31	1	47	1	4	1	1	42	1	0	0	0	0	0	1	0	0	0	1	0	0	1	0	0	0	0	0	0	0	0	0	0
195	32	0	53	1	5	1	1	42	0	0	1	1	1	0	0	0	0	1	1	0	0	1	0	0	0	0	0	0	0	1	0	0
196	33	0	48	1	5	1	1	42	0	0	0	1	1	0	0	0	0	1	1	0	1	1	0	0	0	0	0	0	1	1	0	0
197	34	1	52	1	4	1	1	42	0	0	0	1	0	0	0	0	0	1	1	0	1	0	0	0	0	0	0	0	0	0	0	1
198	35	0	23	1	2	0	0	42	0	0	1	0	1	0	0	0	0	1	0	0	0	1	0	0	0	0	0	1	0	0	0	0
199	36	0	44	0	5	1	1	42	0	1	0	1	0	0	0	0	0	0	0	0	0	1	0	0	0	0	0	0	0	0	1	0
200	37	0	26	1	3	1	1	42	0	1	0	0	0	0	1	0	0	0	0	0	0	0	0	0	0	0	0	1	0	0	0	0
201	38	0	26	1	1	0	0	42	1	0	0	0	0	0	0	1	0	0	0	0	0	0	0	1	0	1	0	0	0	0	0	0

	A	B	C	D	E	F	G	H	I	J	K	L	M	N	O	P	Q	R	S	T	U	V	W	X	Y	Z	AA	AB	AC	AD	AE	AF
1	**Consumer**	Gender 0F 1M	Age (cont.)	Usual consumer 0 no 1 yes	Freshness 1 not fresh 5 very fresh	will to purchase 0 no 1 yes	**will to consume 0 no 1 yes**	**storage time**	Moisten aspect	Slime	Dry	Bright	Cured aspect	Brownish color	Dull	Darker spots	Greenish spots	Wine aroma	garlic aroma	Fermented aroma	Smoke aroma	Cured aroma	Rancid	Sulfur	Mold	Ammoniac/rotten	Butter	Sour/vinegar aroma	Salty tase	Piquant	Sweet taste	Acid taste
202	39	0	44	1	5	1	1	42	1	0	0	1	0	0	0	0	0	1	0	0	1	1	0	0	0	0	0	0	0	0	1	0
203	40	0	21	1	4	1	1	42	0	0	1	0	0	0	1	0	0	0	0	1	0	1	0	0	0	0	0	1	0	0	0	0
204	41	0	28	1	5	1	1	42	0	0	0	1	1	0	0	0	0	0	0	0	0	1	0	0	0	0	0	0	0	0	0	0
205	42	0	24	1	4	1	1	42	0	0	1	0	1	0	0	1	0	1	1	0	1	0	0	0	1	0	0	1	1	0	1	0
206	43	0	23	1	4	1	1	42	0	0	0	1	1	0	0	0	0	0	0	0	0	1	0	0	0	0	0	0	1	0	0	0
207	44	1	32	1	3	0	1	42	0	1	0	0	0	1	0	1	0	0	0	0	0	0	0	0	0	0	0	0	1	0	0	0
208	45	1	55	1	5	1	1	42	0	0	1	1	1	0	0	0	0	1	1	0	0	0	0	0	0	0	0	0	0	0	0	1
209	46	1	54	1	5	1	1	42	0	1	0	0	1	0	0	0	0	0	0	0	0	1	0	0	0	0	0	0	0	1	0	0
210	47	0	57	0	4	0	1	42	1	0	0	0	1	0	0	0	0	0	0	0	0	1	0	0	0	0	0	0	0	0	0	0
211	48	1	42	1	4	0	1	42	1	0	0	1	0	0	0	0	0	0	0	0	0	1	0	0	0	0	0	0	1	0	0	0
212	49	0	26	1	5	1	1	42	0	1	0	1	0	0	0	0	0	1	0	0	1	1	0	0	0	0	1	0	1	0	1	0
213	50	0	56	1	5	1	1	42	1	0	0	0	1	0	0	0	0	1	1	0	0	1	0	0	0	0	0	0	1	1	0	0
214	51	1	29	1	4	1	1	42	1	0	0	1	0	0	0	1	0	0	1	0	1	0	0	0	0	0	0	0	0	0	1	0
215	52	0	26	1	2	0	0	42	0	0	1	0	1	0	0	1	0	0	0	0	0	1	0	0	0	0	0	0	1	0	0	1
216	53	0	26	0	3	0	0	42	1	0	0	1	0	0	0	1	0	1	0	0	1	0	0	0	0	0	0	0	1	0	0	0
217	54	0	60	1	5	1	1	42	1	0	0	0	1	0	0	0	0	1	0	0	1	1	0	0	0	0	0	0	1	0	0	0
218	55	0	50	1	4	1	1	42	0	1	0	1	1	0	0	0	0	0	0	0	0	1	0	0	0	0	0	0	0	0	0	0
219	56	0	54	1	3	1	1	42	0	0	1	0	1	0	1	0	0	1	1	0	1	1	0	0	0	0	0	0	1	1	1	1
220	57	1	38	1	1	0	0	42	0	0	1	0	0	1	0	1	0	1	1	1	1	1	0	0	0	0	0	1	1	0	0	0
221	58	0	24	1	2	0	0	42	0	1	0	1	0	0	0	1	0	0	0	0	1	1	0	0	0	0	0	1	0	0	0	1

	A	B	C	D	E	F	G	H	I	J	K	L	M	N	O	P	Q	R	S	T	U	V	W	X	Y	Z	AA	AB	AC	AD	AE	AF
1	**Consumer**	Gender 0F 1M	Age (cont.)	Usual consumer 0 no 1 yes	Freshness 1 not fresh 5 very fresh	will to purchase 0 no 1 yes	**will to consume 0 no 1 yes**	**storage time**	Moisten aspect	Slime	Dry	Bright	Cured aspect	Brownish color	Dull	Darker spots	Greenish spots	Wine aroma	garlic aroma	Fermented aroma	Smoke aroma	Cured aroma	Rancid	Sulfur	Mold	Ammoniac/rotten	Butter	Sour/vinegar aroma	Salty tase	Piquant	Sweet taste	Acid taste
222	59	0	23	1	3	1	1	42	1	0	0	1	1	0	0	0	0	1	0	0	0	1	1	0	0	0	0	0	1	0	1	1
223	60	1	22	1	4	1	1	42	0	0	1	0	0	0	0	1	0	0	0	0	1	1	0	0	0	0	0	0	1	0	0	0
224	61	1	35	1	4	1	1	42	0	0	1	1	1	0	0	0	0	0	0	0	0	1	0	0	0	0	0	0	0	0	0	0
225	62	1	58	1	4	1	1	42	0	0	1	0	1	0	0	0	0	1	0	0	0	0	0	0	0	0	0	1	1	0	0	0
226	63	0	26	1	3	1	1	42	0	0	1	0	1	0	0	0	0	0	0	0	1	0	1	0	0	0	0	0	0	0	0	0
227	64	0	56	1	4	1	1	42	1	0	0	1	0	0	0	0	0	0	1	0	0	0	0	0	0	0	0	0	0	1	0	0
228	65	0	33	1	3	0	0	42	0	0	1	0	1	0	1	0	0	0	0	0	1	1	0	0	0	0	0	0	1	0	0	1
229	66	0	54	1	3	1	1	42	0	0	1	0	1	0	1	0	0	0	0	0	1	1	0	0	0	0	0	0	0	0	1	0
230	67	1	25	1	3	0	0	42	0	0	1	0	1	0	0	0	0	1	0	0	0	0	0	0	0	0	0	0	1	0	0	0
231	68	1	25	1	5	1	1	42	1	1	0	1	1	1	0	0	0	1	0	0	0	1	0	0	0	0	0	0	1	0	0	0
232	69	1	29	1	4	1	1	42	0	0	1	0	0	0	0	0	0	0	0	0	0	1	0	0	0	0	0	0	0	0	0	0
233	70	1	56	1	2	0	0	42	0	0	0	0	0	0	0	1	0	0	0	1	0	0	0	0	0	0	0	0	1	0	0	0
234	71	1	50	1	4	1	1	42	0	0	1	0	1	0	0	0	0	1	1	1	0	1	0	0	0	0	0	0	1	0	0	1
235	72	0	57	1	4	1	1	42	1	0	0	0	1	0	0	0	0	1	0	0	0	0	0	0	0	0	0	0	1	0	0	0
236	73	1	22	1	4	1	1	42	0	0	1	0	1	0	1	0	0	0	1	0	1	1	0	0	0	0	0	0	1	0	0	0
237	74	1	55	1	5	1	1	42	0	0	1	0	1	0	0	1	0	1	0	0	0	1	0	0	0	0	0	0	0	0	1	0
238	75	0	26	1	4	1	1	42	1	0	0	1	1	0	0	0	0	0	0	0	1	1	0	0	0	0	0	0	1	0	0	0
239	76	0	53	1	4	1	1	42	0	0	1	0	1	0	1	0	0	0	1	0	1	0	0	0	0	0	0	0	1	0	0	0
240	77	1	53	1	4	1	1	42	0	0	1	1	0	0	0	0	0	1	0	0	0	0	0	0	0	0	0	0	1	0	0	0
241	78	1	58	1	4	1	1	42	0	0	1	0	1	0	0	0	0	0	0	0	1	0	0	0	0	0	0	0	1	0	0	0

	A	B	C	D	E	F	G	H	I	J	K	L	M	N	O	P	Q	R	S	T	U	V	W	X	Y	Z	AA	AB	AC	AD	AE	AF
1	**Consumer**	Gender 0F 1M	Age (cont.)	Usual consumer 0 no 1 yes	Freshness 1 not fresh 5 very fresh	will to purchase 0 no 1 yes	**will to consume 0 no 1 yes**	**storage time**	Moisten aspect	Slime	Dry	Bright	Cured aspect	Brownish color	Dull	Darker spots	Greenish spots	Wine aroma	garlic aroma	Fermented aroma	Smoke aroma	Cured aroma	Rancid	Sulfur	Mold	Ammoniac/rotten	Butter	Sour/vinegar aroma	Salty tase	Piquant	Sweet taste	Acid taste
242	79	0	60	1	5	0	1	42	1	0	0	0	1	0	0	0	0	1	1	0	0	0	0	0	0	0	0	0	1	0	0	0
243	80	1	26	1	2	1	1	42	0	0	1	0	0	0	0	1	0	0	0	0	0	1	0	0	0	0	0	0	0	0	0	0
244	81	0	27	1	4	1	1	42	0	1	0	0	0	0	1	0	0	1	0	0	0	0	0	0	0	0	0	0	1	0	0	0
245	1	1	24	1	4	1	**1**	63	**0**	0	1	0	0	0	1	0	0	1	0	0	1	1	0	0	0	0	0	0	1	1	1	0
246	2	0	23	1	1	0	0	63	1	0	0	1	0	0	0	0	0	1	0	0	0	0	1	0	0	0	0	1	0	0	0	1
247	3	1	53	1	4	1	1	63	1	0	0	0	0	0	1	0	0	1	0	0	1	0	0	0	0	0	0	0	0	0	0	0
248	4	0	52	1	3	1	1	63	0	0	1	0	0	0	1	0	0	0	1	0	1	0	0	0	0	0	0	0	1	0	0	1
249	5	0	23	1	2	0	1	63	1	0	0	1	0	0	0	0	0	1	1	0	0	0	0	0	0	0	0	1	0	0	0	0
250	6	0	23	1	2	1	1	63	1	0	0	1	0	0	0	0	0	1	0	0	0	0	0	0	0	0	0	1	1	0	0	1
251	7	1	55	1	3	1	1	63	1	0	0	1	1	0	0	0	0	1	1	0	1	1	0	0	0	0	0	0	1	0	0	1
252	8	0	54	1	3	1	1	63	1	0	0	1	1	0	0	0	0	1	0	0	0	1	0	0	0	0	0	0	1	0	0	0
253	9	1	21	1	4	1	1	63	0	0	1	1	0	0	0	0	0	1	0	0	1	1	0	0	0	0	0	0	1	0	0	0
254	10	1	55	1	4	1	1	63	0	1	0	0	1	0	0	0	0	1	0	0	1	1	0	0	0	0	0	0	0	0	0	1
255	11	0	23	0	4	1	1	63	1	0	0	1	1	0	0	0	0	1	1	0	1	1	0	0	0	0	0	1	1	0	0	1
256	12	0	52	1	5	1	1	63	0	0	1	1	0	0	0	0	0	1	1	0	1	0	0	0	0	0	0	0	1	0	0	0
257	13	1	50	0	4	1	1	63	0	0	1	0	1	0	0	0	0	1	0	0	1	1	0	0	0	0	0	0	1	0	0	0
258	14	1	85	1	4	1	1	63	0	0	1	1	1	0	0	0	0	1	0	0	0	1	0	0	0	0	0	0	0	0	0	0
259	15	1	60	0	3	1	0	63	0	0	1	0	1	0	0	0	0	0	0	0	1	0	0	0	0	0	0	0	1	0	0	0
260	16	0	58	1	3	0	1	63	0	0	1	1	1	0	0	0	0	0	0	0	1	1	0	0	0	0	0	0	0	0	0	1
261	17	1	30	1	5	1	1	63	0	0	1	1	1	0	0	0	0	0	1	0	0	1	0	0	0	0	0	0	1	0	0	0

	A	B	C	D	E	F	G	H	I	J	K	L	M	N	O	P	Q	R	S	T	U	V	W	X	Y	Z	AA	AB	AC	AD	AE	AF
1	**Consumer**	Gender 0F 1M	Age (cont.)	Usual consumer 0 no 1 yes	Freshness 1 not fresh 5 very fresh	will to purchase 0 no 1 yes	**will to consume 0 no 1 yes**	**storage time**	Moisten aspect	Slime	Dry	Bright	Cured aspect	Brownish color	Dull	Darker spots	Greenish spots	Wine aroma	garlic aroma	Fermented aroma	Smoke aroma	Cured aroma	Rancid	Sulfur	Mold	Ammoniac/rotten	Butter	Sour/vinegar aroma	Salty tase	Piquant	Sweet taste	Acid taste
262	18	0	59	1	5	1	1	63	0	0	1	0	1	0	0	0	0	1	1	0	0	1	0	0	0	0	0	0	1	0	0	0
263	19	0	23	1	3	1	1	63	0	0	1	0	0	0	1	0	0	0	0	0	0	1	0	0	0	0	0	0	0	0	0	1
264	20	0	22	0	2	0	0	63	0	0	1	0	1	0	1	0	0	0	0	0	1	1	0	0	0	0	0	1	1	0	0	0
265	21	0	23	1	5	1	1	63	0	0	1	0	1	0	0	0	0	1	1	0	1	1	0	0	0	0	0	0	1	0	0	0
266	22	0	28	1	4	1	1	63	0	0	1	0	1	0	0	0	0	0	0	0	0	1	0	0	0	0	0	1	0	0	0	0
267	23	1	23	1	4	1	1	63	1	0	0	0	1	0	0	0	0	1	1	0	0	1	0	0	0	0	0	0	1	0	0	0
268	24	1	39	1	3	1	1	63	1	0	0	1	1	0	1	0	0	1	1	1	0	1	0	0	0	0	0	0	1	0	0	0
269	25	0	26	1	4	1	1	63	1	0	0	0	1	0	0	0	0	1	1	0	0	1	0	0	0	0	0	0	1	0	0	1
270	26	0	27	1	4	1	1	63	0	0	1	1	0	0	0	1	0	1	1	0	1	1	0	0	0	0	0	0	1	1	0	1
271	27	0	36	1	4	1	1	63	1	0	0	1	1	0	0	0	0	1	1	0	0	1	0	0	0	0	0	0	0	0	0	0
272	28	1	49	1	4	1	1	63	1	0	0	1	1	1	0	0	0	1	1	0	0	0	0	0	0	0	0	0	0	0	0	0
273	29	1	23	0	4	1	1	63	0	1	1	0	1	0	1	0	0	0	0	0	0	1	0	0	0	0	0	0	0	0	0	1
274	30	0	46	1	4	1	1	63	0	0	1	0	0	0	1	0	0	1	0	0	1	1	0	0	0	0	0	0	1	0	0	0
275	31	1	47	1	4	1	1	63	0	1	0	0	1	0	0	0	0	1	0	0	0	1	0	0	0	0	0	0	0	1	0	0
276	32	0	53	1	5	1	1	63	0	0	1	0	1	0	1	0	0	1	1	0	0	1	0	0	0	0	0	0	0	1	0	0
277	33	0	48	1	4	0	1	63	0	0	1	0	1	0	1	0	0	1	0	0	1	1	0	0	0	0	0	0	0	0	0	0
278	34	1	52	1	5	1	1	63	0	0	0	0	0	0	0	0	0	1	0	0	1	1	0	0	0	0	0	0	0	0	0	0
279	35	0	23	1	4	1	1	63	0	0	1	1	1	0	0	0	0	1	0	0	1	1	0	0	0	0	0	0	1	0	0	0
280	36	0	44	0	5	1	1	63	0	0	1	0	1	0	0	0	0	0	0	0	0	1	0	0	0	0	0	0	0	0	1	0
281	37	0	26	1	3	0	0	63	0	0	1	0	0	0	1	0	0	0	0	0	0	0	0	0	0	0	0	1	0	0	0	0

	A	B	C	D	E	F	G	H	I	J	K	L	M	N	O	P	Q	R	S	T	U	V	W	X	Y	Z	AA	AB	AC	AD	AE	AF
1	**Consumer**	Gender 0F 1M	Age (cont.)	Usual consumer 0 no 1 yes	Freshness 1 not fresh 5 very fresh	will to purchase 0 no 1 yes	**will to consume 0 no 1 yes**	**storage time**	Moisten aspect	Slime	Dry	Bright	Cured aspect	Brownish color	Dull	Darker spots	Greenish spots	Wine aroma	garlic aroma	Fermented aroma	Smoke aroma	Cured aroma	Rancid	Sulfur	Mold	Ammoniac/rotten	Butter	Sour/vinegar aroma	Salty tase	Piquant	Sweet taste	Acid taste
282	38	0	26	1	4	1	1	63	1	0	0	0	1	0	0	0	0	0	1	0	1	1	0	0	0	0	0	0	0	0	0	0
283	39	0	44	1	5	0	1	63	0	0	1	0	1	0	1	0	0	1	0	0	0	1	0	0	0	0	0	0	1	0	0	0
284	40	0	21	1	4	1	1	63	0	0	1	0	1	0	0	0	0	0	1	0	0	1	0	0	0	0	0	0	0	0	1	0
285	41	0	28	1	4	1	1	63	0	0	0	0	1	0	1	0	0	0	0	0	0	1	0	0	0	0	0	0	0	1	1	0
286	42	0	24	1	3	0	0	63	0	0	1	0	1	0	1	1	0	1	1	0	1	1	0	0	0	0	0	0	1	0	1	0
287	43	0	23	1	4	1	1	63	1	0	0	0	1	0	0	0	0	0	0	0	0	0	0	0	0	0	0	0	1	0	0	0
288	44	1	32	1	2	0	0	63	0	0	1	0	0	1	0	1	0	0	0	0	0	0	0	0	0	0	0	0	1	0	0	0
289	45	1	55	1	5	1	1	63	0	0	1	0	1	0	0	0	0	1	0	0	0	1	0	0	0	0	0	0	1	0	0	1
290	46	1	54	1	4	0	0	63	0	1	0	0	1	0	0	0	0	1	0	0	0	0	0	0	0	0	1	0	0	0	0	0
291	47	0	57	0	3	0	0	63	0	0	1	0	1	0	0	0	0	0	0	0	0	1	0	0	0	0	0	1	0	0	0	0
292	48	1	42	1	2	0	0	63	0	0	1	0	1	0	0	0	0	0	0	0	0	1	0	0	0	0	0	0	0	0	0	0
293	49	0	26	1	4	1	1	63	0	1	0	1	1	0	0	0	0	1	1	0	0	1	0	0	0	0	0	0	1	0	1	1
294	50	0	56	1	5	1	1	63	1	0	0	0	1	0	0	0	0	1	1	0	0	1	0	0	0	0	0	0	0	0	0	0
295	51	1	29	1	4	1	1	63	0	0	1	0	1	0	1	0	0	1	1	0	0	1	0	0	0	0	0	0	1	0	0	0
296	52	0	26	1	4	1	1	63	0	0	1	0	0	0	1	1	0	0	1	0	1	0	0	0	0	0	0	0	1	0	0	0
297	53	0	26	0	3	0	0	63	0	0	1	0	1	0	0	1	0	0	0	0	0	1	0	0	0	0	0	0	0	0	0	0
298	54	0	60	1	3	0	0	63	1	0	0	1	0	1	0	0	0	1	1	0	0	1	0	0	0	0	0	0	1	0	0	0
299	55	0	50	1	5	1	1	63	0	0	1	0	1	0	0	0	0	1	0	0	1	1	0	0	0	0	0	0	1	0	0	0
300	56	0	54	1	4	1	1	63	0	1	0	0	1	0	1	0	0	0	0	0	0	1	0	0	0	0	0	0	0	0	0	0
301	57	1	38	1	3	1	1	63	0	0	1	0	1	0	1	0	0	1	1	0	1	1	0	0	0	0	0	0	1	0	1	1

	A	B	C	D	E	F	G	H	I	J	K	L	M	N	O	P	Q	R	S	T	U	V	W	X	Y	Z	AA	AB	AC	AD	AE	AF
1	**Consumer**	Gender 0F 1M	Age (cont.)	Usual consumer 0 no 1 yes	Freshness 1 not fresh 5 very fresh	will to purchase 0 no 1 yes	**will to consume 0 no 1 yes**	**storage time**	Moisten aspect	Slime	Dry	Bright	Cured aspect	Brownish color	Dull	Darker spots	Greenish spots	Wine aroma	garlic aroma	Fermented aroma	Smoke aroma	Cured aroma	Rancid	Sulfur	Mold	Ammoniac/rotten	Butter	Sour/vinegar aroma	Salty tase	Piquant	Sweet taste	Acid taste
302	58	0	24	1	1	0	0	63	0	0	1	0	0	0	1	0	0	1	1	1	0	0	0	0	0	0	0	0	0	1	0	0
303	59	0	23	1	2	0	0	63	0	0	1	0	0	0	1	1	0	0	0	1	0	1	0	0	0	0	0	1	0	0	0	0
304	60	1	22	1	2	0	1	63	0	0	1	0	1	0	1	0	0	0	0	1	0	1	1	0	0	1	0	1	0	0	1	0
305	61	1	35	1	3	0	1	63	1	0	0	1	0	0	0	0	0	0	0	0	0	0	0	0	0	0	1	0	1	0	0	0
306	62	1	58	1	4	1	1	63	0	0	1	1	1	0	0	0	0	0	0	0	0	1	0	0	0	0	0	0	0	0	0	0
307	63	0	26	1	4	1	1	63	0	0	1	1	1	0	0	0	0	1	1	0	0	0	0	0	0	0	0	0	1	0	0	0
308	64	0	56	1	3	1	1	63	0	0	1	0	1	0	0	0	0	1	1	0	1	1	0	0	0	0	0	0	1	0	0	0
309	65	0	33	1	3	0	0	63	0	0	1	0	0	1	0	0	0	0	0	1	0	0	0	0	0	0	0	1	0	0	0	0
310	66	0	54	1	4	1	1	63	0	0	1	1	0	0	0	0	0	1	1	0	0	1	0	0	0	0	0	0	0	0	1	0
311	67	1	25	1	4	1	1	63	1	0	0	1	1	0	0	0	0	0	0	0	0	1	0	0	0	0	0	0	0	0	1	0
312	68	1	25	1	3	0	0	63	0	0	1	0	1	0	0	0	0	0	0	0	0	1	0	0	0	0	0	0	1	0	0	0
313	69	1	29	1	4	1	1	63	1	1	0	1	1	1	0	0	0	1	0	0	0	1	0	0	0	0	0	0	1	0	0	0
314	70	1	56	1	2	0	0	63	1	0	0	1	0	0	0	0	0	0	0	0	0	0	0	0	0	0	0	0	1	0	0	0
315	71	1	50	1	2	1	1	63	0	0	0	0	1	0	0	0	0	0	0	0	0	1	0	0	0	0	0	1	0	0	0	0
316	72	0	57	1	4	1	1	63	0	0	1	0	1	0	0	0	0	1	0	1	0	1	0	0	0	0	0	0	1	0	0	1
317	73	1	22	1	4	1	1	63	1	0	0	0	1	0	0	0	0	1	0	0	0	0	0	0	0	0	0	0	1	0	0	0
318	74	1	55	1	3	0	1	63	0	0	1	0	0	0	1	0	0	1	1	0	1	0	0	0	0	0	0	0	1	0	0	0
319	75	0	26	1	5	1	1	63	0	0	1	0	1	0	0	0	0	1	1	0	0	0	0	0	0	0	0	0	1	0	0	0
320	76	0	53	1	4	1	1	63	0	0	1	1	1	0	0	0	0	0	0	0	1	0	0	0	0	0	0	0	0	0	1	0
321	77	1	53	1	4	1	1	63	0	0	1	0	0	0	1	0	0	0	0	0	1	1	0	0	0	0	0	0	0	0	0	1

	A	B	C	D	E	F	G	H	I	J	K	L	M	N	O	P	Q	R	S	T	U	V	W	X	Y	Z	AA	AB	AC	AD	AE	AF
1	**Consumer**	Gender 0F 1M	Age (cont.)	Usual consumer 0 no 1 yes	Freshness 1 not fresh 5 very fresh	will to purchase 0 no 1 yes	**will to consume 0 no 1 yes**	**storage time**	Moisten aspect	Slime	Dry	Bright	Cured aspect	Brownish color	Dull	Darker spots	Greenish spots	Wine aroma	garlic aroma	Fermented aroma	Smoke aroma	Cured aroma	Rancid	Sulfur	Mold	Ammoniac/rotten	Butter	Sour/vinegar aroma	Salty tase	Piquant	Sweet taste	Acid taste
322	78	1	58	1	4	1	1	63	0	0	1	0	0	0	0	1	0	0	0	0	1	0	0	0	0	0	0	0	0	0	0	0
323	79	0	60	1	4	1	1	63	0	0	0	0	0	0	0	1	0	1	0	0	0	0	0	0	0	0	0	0	1	0	0	0
324	80	1	26	1	5	0	0	63	0	0	1	0	1	0	0	0	0	1	0	0	0	0	0	0	0	0	0	0	1	0	0	0
325	81	0	27	1	3	1	1	63	0	1	0	0	1	0	0	0	0	0	0	0	0	1	0	0	0	0	0	0	0	0	0	0
326	1	1	24	1	2	0	**0**	84	**1**	0	0	0	0	1	0	1	0	1	1	0	1	1	0	0	0	0	0	0	1	1	0	0
327	2	0	23	1	5	0	1	84	0	0	1	0	1	0	0	0	0	1	1	0	0	0	0	0	0	0	0	0	1	0	1	0
328	3	1	53	1	5	1	1	84	1	0	0	1	1	0	0	0	0	0	1	0	1	1	0	0	0	0	0	0	1	1	0	0
329	4	0	52	1	4	1	1	84	0	1	0	0	0	0	0	0	0	0	0	0	1	1	0	0	0	0	0	0	0	0	0	0
330	5	0	23	1	1	0	0	84	1	1	0	1	1	0	1	0	0	0	0	0	0	0	0	0	0	0	0	0	1	0	0	1
331	6	0	23	1	4	1	1	84	0	0	1	1	1	0	0	0	0	1	1	0	0	1	0	0	0	0	0	0	1	0	0	1
332	7	1	55	1	5	0	0	84	1	0	0	0	1	0	0	0	0	1	1	0	0	0	0	0	0	0	0	0	1	0	0	0
333	8	0	54	1	4	1	1	84	0	0	1	0	0	1	0	0	0	0	1	0	0	0	0	0	0	0	0	0	1	0	0	0
334	9	1	21	1	5	1	1	84	0	1	0	0	1	0	0	0	0	1	0	0	0	0	0	0	0	0	0	0	0	0	0	0
335	10	1	55	1	4	1	1	84	0	0	1	0	1	0	0	0	0	1	0	0	0	1	0	0	0	0	0	0	0	0	0	0
336	11	0	23	0	4	1	1	84	1	0	0	1	0	0	0	0	0	0	0	0	1	1	0	0	0	0	0	0	0	0	1	0
337	12	0	52	1	3	0	1	84	0	0	1	0	0	0	1	1	0	0	0	1	0	0	0	0	0	0	0	1	0	0	0	0
338	13	1	50	0	3	1	1	84	0	0	1	0	0	1	1	0	0	1	0	0	0	1	0	0	0	0	0	0	0	0	0	0
339	14	1	85	1	3	1	1	84	0	0	1	0	1	0	0	0	0	0	1	0	1	1	0	0	0	0	0	0	1	0	0	0
340	15	1	60	0	4	1	1	84	1	0	0	0	1	0	0	0	0	1	0	0	0	0	0	0	0	0	0	0	1	0	0	0
341	16	0	58	1	4	1	1	84	0	0	1	0	1	0	0	0	0	1	1	1	1	1	0	0	0	0	0	1	1	0	0	1

	A	B	C	D	E	F	G	H	I	J	K	L	M	N	O	P	Q	R	S	T	U	V	W	X	Y	Z	AA	AB	AC	AD	AE	AF
1	**Consumer**	Gender 0F 1M	Age (cont.)	Usual consumer 0 no 1 yes	Freshness 1 not fresh 5 very fresh	will to purchase 0 no 1 yes	**will to consume 0 no 1 yes**	**storage time**	Moisten aspect	Slime	Dry	Bright	Cured aspect	Brownish color	Dull	Darker spots	Greenish spots	Wine aroma	garlic aroma	Fermented aroma	Smoke aroma	Cured aroma	Rancid	Sulfur	Mold	Ammoniac/rotten	Butter	Sour/vinegar aroma	Salty tase	Piquant	Sweet taste	Acid taste
342	17	1	30	1	3	1	1	84	0	0	0	0	1	0	0	0	0	1	0	0	0	0	0	0	0	0	0	0	0	0	0	0
343	18	0	59	1	2	1	1	84	1	0	0	1	0	0	0	0	0	0	0	0	0	1	0	0	0	0	0	0	0	0	0	0
344	19	0	23	1	4	1	1	84	0	0	0	0	1	1	1	0	0	1	0	0	0	1	0	0	0	0	0	0	0	0	0	0
345	20	0	22	0	2	0	0	84	1	0	0	0	1	0	0	0	0	0	0	0	0	1	0	0	0	0	0	0	0	0	0	0
346	21	0	23	1	3	1	1	84	0	0	1	0	1	0	1	0	0	0	0	1	0	1	0	0	0	0	0	0	0	0	1	0
347	22	0	28	1	4	1	1	84	0	0	1	1	1	0	0	0	0	1	0	0	0	1	0	0	0	0	0	0	0	0	0	0
348	23	1	23	1	2	0	0	84	0	0	1	0	0	0	1	0	0	0	0	1	0	0	0	0	0	0	0	0	0	1	0	0
349	24	1	39	1	3	1	1	84	0	0	1	0	1	0	0	0	0	1	1	0	0	1	0	0	0	0	0	0	1	0	0	1
350	25	0	26	1	3	1	1	84	0	0	1	0	0	0	1	0	0	1	0	0	1	0	0	0	0	0	0	1	0	0	0	0
351	26	0	27	1	4	1	1	84	0	0	1	0	1	0	0	0	0	0	0	0	0	1	0	0	0	0	0	0	0	0	0	0
352	27	0	36	1	3	1	1	84	1	0	0	1	1	0	0	0	0	1	0	0	0	1	0	0	0	0	0	1	0	0	1	0
353	28	1	49	1	2	1	1	84	0	0	1	1	1	0	0	0	0	1	0	0	1	0	0	0	0	0	0	1	0	0	0	0
354	29	1	23	0	1	0	0	84	0	0	1	0	0	0	1	0	0	1	1	1	0	0	1	0	0	0	0	1	1	1	0	0
355	30	0	46	1	3	1	1	84	0	0	1	0	1	0	1	0	0	1	1	1	1	1	0	0	0	0	0	0	1	1	0	1
356	31	1	47	1	3	1	1	84	0	0	1	0	1	0	0	0	1	0	0	0	0	1	0	0	0	0	0	0	0	0	0	0
357	32	0	53	1	4	1	1	84	0	0	1	0	1	0	0	0	0	1	0	0	1	1	0	0	0	0	0	0	1	0	0	0
358	33	0	48	1	3	1	1	84	1	0	0	1	0	0	0	0	0	1	0	0	1	0	0	0	0	0	0	0	0	0	0	0
359	34	1	52	1	3	0	1	84	0	0	1	0	0	0	0	1	1	1	0	0	1	0	0	0	0	0	0	0	0	0	0	0
360	35	0	23	1	5	1	1	84	0	0	0	0	1	0	0	0	0	0	0	0	0	0	0	0	0	0	0	0	0	0	0	0
361	36	0	44	0	3	0	1	84	0	0	1	0	0	1	1	0	0	1	0	0	0	1	0	0	0	0	0	1	1	0	0	0

	A	B	C	D	E	F	G	H	I	J	K	L	M	N	O	P	Q	R	S	T	U	V	W	X	Y	Z	AA	AB	AC	AD	AE	AF
1	**Consumer**	Gender 0F 1M	Age (cont.)	Usual consumer 0 no 1 yes	Freshness 1 not fresh 5 very fresh	will to purchase 0 no 1 yes	**will to consume 0 no 1 yes**	**storage time**	Moisten aspect	Slime	Dry	Bright	Cured aspect	Brownish color	Dull	Darker spots	Greenish spots	Wine aroma	garlic aroma	Fermented aroma	Smoke aroma	Cured aroma	Rancid	Sulfur	Mold	Ammoniac/rotten	Butter	Sour/vinegar aroma	Salty tase	Piquant	Sweet taste	Acid taste
362	37	0	26	1	4	0	0	84	1	0	0	1	0	0	0	0	0	0	0	1	0	0	0	0	0	0	0	0	0	0	0	0
363	38	0	26	1	3	0	1	84	1	0	0	0	1	0	0	0	0	1	0	0	0	0	0	0	0	0	0	0	0	0	0	0
364	39	0	44	1	5	1	1	84	0	1	0	0	1	0	0	0	0	1	0	0	0	0	0	0	0	0	0	0	0	1	0	0
365	40	0	21	1	5	1	1	84	0	0	1	0	1	0	0	0	0	0	0	0	0	1	0	0	0	0	0	0	1	0	0	0
366	41	0	28	1	5	1	1	84	0	1	0	1	0	0	0	0	0	0	0	0	0	0	0	0	0	0	0	0	1	0	0	0
367	42	0	24	1	4	1	1	84	1	0	0	1	1	0	0	0	0	0	1	0	0	0	0	0	0	0	0	0	1	0	0	0
368	43	0	23	1	4	1	1	84	0	0	1	1	1	0	0	0	1	1	0	1	0	1	0	0	0	0	0	0	0	1	1	0
369	44	1	32	1	4	1	1	84	1	0	0	1	0	0	0	0	0	1	0	0	0	0	0	0	0	0	0	1	0	0	0	0
370	45	1	55	1	5	1	1	84	1	0	0	1	1	0	0	0	0	0	0	0	0	1	0	0	0	0	0	0	1	0	0	0
371	46	1	54	1	3	0	0	84	0	1	0	0	0	0	0	1	0	0	0	0	0	0	1	0	0	0	0	0	0	0	0	1
372	47	0	57	0	3	1	1	84	0	1	0	0	0	1	0	0	0	1	1	0	0	0	0	0	0	0	0	0	0	0	0	0
373	48	1	42	1	5	1	1	84	1	0	0	1	0	0	0	0	0	0	0	0	0	0	0	0	0	0	0	0	1	0	0	1
374	49	0	26	1	2	0	0	84	0	0	1	0	1	0	0	1	0	0	0	0	0	0	0	0	0	0	0	1	0	0	0	1
375	50	0	56	1	5	1	1	84	0	0	0	1	1	1	0	0	0	1	1	0	1	0	0	0	0	0	0	0	0	1	0	0
376	51	1	29	1	3	0	0	84	1	0	0	1	1	0	0	0	0	1	0	0	1	1	0	0	0	0	0	0	0	0	0	0
377	52	0	26	1	5	1	1	84	0	0	1	0	1	0	1	0	0	1	1	0	0	1	0	0	0	0	0	0	0	0	0	0
378	53	0	26	0	5	1	1	84	1	0	0	0	1	0	0	0	0	0	1	0	0	1	0	0	0	0	0	0	0	0	0	1
379	54	0	60	1	5	1	1	84	1	0	0	1	1	0	0	0	0	1	1	0	1	1	0	0	0	0	0	1	1	0	0	0
380	55	0	50	1	3	0	1	84	1	1	0	1	0	0	0	1	0	0	0	1	0	1	0	0	0	0	0	1	1	0	0	1
381	56	0	54	1	4	1	1	84	1	0	0	1	1	1	0	0	0	1	1	0	0	0	0	0	0	0	0	0	0	0	0	0

	A	B	C	D	E	F	G	H	I	J	K	L	M	N	O	P	Q	R	S	T	U	V	W	X	Y	Z	AA	AB	AC	AD	AE	AF
1	**Consumer**	Gender 0F 1M	Age (cont.)	Usual consumer 0 no 1 yes	Freshness 1 not fresh 5 very fresh	will to purchase 0 no 1 yes	**will to consume 0 no 1 yes**	**storage time**	Moisten aspect	Slime	Dry	Bright	Cured aspect	Brownish color	Dull	Darker spots	Greenish spots	Wine aroma	garlic aroma	Fermented aroma	Smoke aroma	Cured aroma	Rancid	Sulfur	Mold	Ammoniac/rotten	Butter	Sour/vinegar aroma	Salty tase	Piquant	Sweet taste	Acid taste
382	57	1	38	1	4	1	1	84	1	0	0	0	1	0	1	0	0	1	1	0	0	1	0	0	0	0	0	0	0	0	0	0
383	58	0	24	1	4	1	1	84	1	0	0	0	1	0	0	0	0	1	1	0	0	1	0	0	0	0	0	1	1	0	0	1
384	59	0	23	1	3	1	1	84	1	0	0	1	1	0	1	0	0	1	1	0	1	1	0	0	0	0	0	0	1	0	0	0
385	60	1	22	1	3	1	1	84	0	1	0	0	1	0	0	0	0	1	0	0	0	1	0	0	0	0	0	0	1	0	0	0
386	61	1	35	1	1	0	0	84	0	0	1	0	0	0	0	1	0	0	0	0	0	0	0	0	0	0	0	0	1	0	0	1
387	62	1	58	1	3	1	1	84	1	0	0	0	0	0	0	1	0	1	0	0	1	0	0	0	0	0	0	0	0	0	0	1
388	63	0	26	1	3	0	0	84	1	0	0	0	0	0	1	0	0	1	0	0	0	0	0	0	0	0	0	1	0	0	0	0
389	64	0	56	1	4	1	1	84	0	0	1	0	1	0	0	0	0	0	0	0	1	0	0	0	0	0	0	0	0	1	0	0
390	65	0	33	1	3	1	1	84	1	0	0	1	1	0	0	0	0	1	0	1	0	0	0	0	0	0	0	1	1	0	0	1
391	66	0	54	1	4	1	1	84	0	0	1	0	1	0	0	0	0	1	0	0	1	0	0	0	0	0	0	0	1	0	0	0
392	67	1	25	1	5	1	1	84	1	0	0	0	1	0	0	0	0	0	0	0	0	1	0	0	0	0	0	1	1	0	0	1
393	68	1	25	1	4	1	1	84	1	0	0	1	1	0	0	0	0	1	1	0	0	1	0	0	0	0	0	0	1	0	0	0
394	69	1	29	1	5	1	1	84	1	0	0	1	0	0	0	0	0	1	1	0	1	1	0	0	0	0	0	0	1	0	0	0
395	70	1	56	1	4	1	1	84	1	0	0	1	1	0	0	0	0	1	1	0	0	0	0	0	0	0	0	0	1	0	0	0
396	71	1	50	1	3	1	1	84	1	0	1	0	1	0	0	0	0	0	0	0	1	0	0	0	0	0	0	0	1	0	0	0
397	72	0	57	1	5	1	1	84	0	1	0	1	1	0	0	0	0	1	1	0	0	0	0	0	0	0	0	0	1	0	0	0
398	73	1	22	1	5	1	1	84	0	0	1	0	1	0	0	0	0	1	1	0	0	1	0	0	0	0	0	0	1	0	0	1
399	74	1	55	1	4	1	1	84	1	0	0	1	1	0	0	0	0	1	1	0	0	0	0	0	0	0	0	0	1	0	0	0
400	75	0	26	1	4	1	1	84	0	0	1	0	1	0	0	0	0	0	1	0	0	1	0	0	0	0	0	0	0	0	1	0
401	76	0	53	1	4	1	1	84	1	0	0	1	1	0	0	0	0	1	1	0	0	1	0	0	0	0	0	0	1	0	0	0

	A	B	C	D	E	F	G	H	I	J	K	L	M	N	O	P	Q	R	S	T	U	V	W	X	Y	Z	AA	AB	AC	AD	AE	AF
1	**Consumer**	Gender 0F 1M	Age (cont.)	Usual consumer 0 no 1 yes	Freshness 1 not fresh 5 very fresh	will to purchase 0 no 1 yes	**will to consume 0 no 1 yes**	**storage time**	Moisten aspect	Slime	Dry	Bright	Cured aspect	Brownish color	Dull	Darker spots	Greenish spots	Wine aroma	garlic aroma	Fermented aroma	Smoke aroma	Cured aroma	Rancid	Sulfur	Mold	Ammoniac/rotten	Butter	Sour/vinegar aroma	Salty tase	Piquant	Sweet taste	Acid taste
402	77	1	53	1	3	1	1	84	0	0	1	0	0	0	1	0	0	1	0	0	1	0	0	0	0	0	0	0	1	1	0	0
403	78	1	58	1	3	1	1	84	1	0	0	1	0	0	0	0	0	0	1	0	0	0	0	0	0	0	0	0	1	0	0	0
404	79	0	60	1	4	1	1	84	0	1	0	0	0	0	1	0	0	0	0	0	0	0	0	0	0	0	0	0	1	0	0	0
405	80	1	26	1	3	0	1	84	0	0	1	1	0	0	0	0	0	0	0	0	0	0	0	0	0	0	0	0	1	0	0	0
406	81	0	27	1	5	1	1	84	0	0	1	1	1	0	0	0	0	1	0	0	0	1	0	0	0	0	0	0	1	0	0	0
407	1	1	24	1	5	1	**1**	105	**0**	0	1	0	1	0	1	0	0	1	1	0	0	1	0	0	0	0	0	0	1	0	0	0
408	2	0	23	1	4	1	1	105	0	0	1	0	1	0	0	0	0	0	0	0	0	0	0	0	0	0	0	0	1	0	0	0
409	3	1	53	1	4	1	1	105	1	0	0	0	0	0	0	1	0	1	0	0	0	1	0	0	0	0	0	0	1	0	0	0
410	4	0	52	1	3	1	1	105	0	0	1	1	1	0	0	0	0	1	1	0	1	1	0	0	0	0	0	0	1	0	0	0
411	5	0	23	1	4	1	1	105	1	0	0	1	1	0	0	0	0	1	1	1	0	1	0	0	0	0	0	0	1	0	0	1
412	6	0	23	1	4	1	1	105	0	1	1	1	1	0	0	1	0	1	1	0	1	1	0	0	0	0	0	0	1	0	0	1
413	7	1	55	1	5	1	1	105	1	0	0	1	1	0	0	0	0	0	1	0	1	1	0	0	0	0	0	0	0	0	0	0
414	8	0	54	1	4	1	1	105	1	0	0	1	1	1	0	0	0	1	1	0	0	0	0	0	0	0	0	0	0	0	0	0
415	9	1	21	1	3	1	1	105	0	1	1	1	0	0	1	0	0	0	0	0	0	1	0	0	0	0	0	0	1	0	0	1
416	10	1	55	1	4	1	1	105	1	0	0	0	1	0	1	0	0	1	1	0	1	1	0	0	0	0	0	1	1	0	0	0
417	11	0	23	0	4	1	1	105	0	1	0	0	1	0	0	0	0	0	0	0	0	1	0	0	0	0	0	0	0	0	0	0
418	12	0	52	1	5	1	1	105	0	0	1	1	1	0	0	0	0	1	1	0	0	1	0	0	0	0	0	0	0	1	0	0
419	13	1	50	0	4	1	1	105	0	0	1	1	1	0	0	0	0	0	0	0	0	1	0	0	0	0	0	0	0	0	0	0
420	14	1	85	1	5	1	1	105	0	0	0	1	0	0	0	0	0	1	1	0	1	1	0	0	0	0	0	0	1	0	0	0
421	15	1	60	0	4	1	1	105	0	0	1	1	1	0	0	0	0	1	1	0	1	1	0	0	0	0	0	0	0	0	0	0

	A	B	C	D	E	F	G	H	I	J	K	L	M	N	O	P	Q	R	S	T	U	V	W	X	Y	Z	AA	AB	AC	AD	AE	AF
1	**Consumer**	Gender 0F 1M	Age (cont.)	Usual consumer 0 no 1 yes	Freshness 1 not fresh 5 very fresh	will to purchase 0 no 1 yes	**will to consume 0 no 1 yes**	**storage time**	Moisten aspect	Slime	Dry	Bright	Cured aspect	Brownish color	Dull	Darker spots	Greenish spots	Wine aroma	garlic aroma	Fermented aroma	Smoke aroma	Cured aroma	Rancid	Sulfur	Mold	Ammoniac/rotten	Butter	Sour/vinegar aroma	Salty tase	Piquant	Sweet taste	Acid taste
422	16	0	58	1	5	1	1	105	0	0	1	0	1	0	0	0	0	0	0	0	0	1	0	0	0	0	0	0	0	0	0	1
423	17	1	30	1	3	1	1	105	0	1	0	0	0	1	0	0	0	1	1	0	0	0	0	0	0	0	0	0	0	0	0	0
424	18	0	59	1	2	0	0	105	0	1	0	0	0	1	0	0	0	0	0	0	0	0	1	1	0	0	0	0	0	1	0	1
425	19	0	23	1	5	0	0	105	0	0	1	1	0	0	0	0	0	1	0	0	1	0	0	0	0	0	0	0	1	0	0	1
426	20	0	22	0	4	1	1	105	0	1	0	0	0	1	0	0	0	1	1	0	0	0	0	0	0	0	0	0	1	0	0	0
427	21	0	23	1	1	0	0	105	0	0	1	0	1	1	1	0	0	0	0	0	0	0	0	0	0	0	0	1	0	0	0	0
428	22	0	28	1	4	1	1	105	1	0	0	1	1	0	0	1	0	1	1	0	1	0	0	0	0	0	0	0	1	0	1	0
429	23	1	23	1	4	1	1	105	0	0	1	1	1	0	0	0	0	0	0	0	0	1	0	0	0	0	0	0	1	0	0	0
430	24	1	39	1	4	1	1	105	0	1	0	0	0	1	0	0	0	0	0	0	0	0	0	0	0	0	0	1	1	0	0	0
431	25	0	26	1	5	1	1	105	0	0	1	0	1	0	0	0	0	1	1	0	0	1	0	0	0	0	0	0	0	0	0	1
432	26	0	27	1	4	1	1	105	0	0	1	0	0	0	1	0	0	1	0	0	0	0	0	0	0	0	0	0	0	1	0	0
433	27	0	36	1	2	0	0	105	0	0	1	0	1	0	0	0	0	0	0	0	0	1	0	0	0	0	0	0	1	0	0	0
434	28	1	49	1	3	0	0	105	0	0	1	0	1	0	0	0	0	0	0	0	0	1	0	0	0	0	0	0	1	0	0	0
435	29	1	23	0	5	1	1	105	0	1	1	0	1	0	1	0	0	1	0	0	1	1	0	0	0	0	0	0	1	0	1	1
436	30	0	46	1	5	1	1	105	1	0	0	1	1	0	0	0	0	1	0	0	1	0	0	0	0	0	0	0	0	1	0	0
437	31	1	47	1	4	0	1	105	0	0	1	0	1	0	0	0	0	1	0	1	0	0	0	0	0	0	0	0	0	1	0	0
438	32	0	53	1	4	1	1	105	0	1	0	1	1	0	0	0	0	0	1	0	0	1	0	0	0	0	0	0	1	0	0	0
439	33	0	48	1	4	0	0	105	1	0	0	1	0	0	0	0	0	1	0	0	0	1	0	0	0	0	0	0	1	0	0	0
440	34	1	52	1	5	1	1	105	0	0	1	0	1	0	0	0	0	1	0	1	1	0	0	0	0	0	0	0	1	0	0	0
441	35	0	23	1	4	1	1	105	0	1	0	1	1	0	0	0	0	0	0	0	0	1	0	0	0	0	0	0	0	0	0	0

	A	B	C	D	E	F	G	H	I	J	K	L	M	N	O	P	Q	R	S	T	U	V	W	X	Y	Z	AA	AB	AC	AD	AE	AF
1	**Consumer**	Gender 0F 1M	Age (cont.)	Usual consumer 0 no 1 yes	Freshness 1 not fresh 5 very fresh	will to purchase 0 no 1 yes	**will to consume 0 no 1 yes**	**storage time**	Moisten aspect	Slime	Dry	Bright	Cured aspect	Brownish color	Dull	Darker spots	Greenish spots	Wine aroma	garlic aroma	Fermented aroma	Smoke aroma	Cured aroma	Rancid	Sulfur	Mold	Ammoniac/rotten	Butter	Sour/vinegar aroma	Salty tase	Piquant	Sweet taste	Acid taste
442	36	0	44	0	3	1	1	105	0	0	1	0	1	0	1	0	0	1	1	0	1	1	0	0	0	0	0	0	1	1	1	1
443	37	0	26	1	1	0	0	105	0	0	1	0	0	0	1	0	0	1	1	1	1	0	0	0	0	0	0	1	1	0	0	0
444	38	0	26	1	1	0	0	105	0	0	1	0	1	1	1	1	0	0	0	0	1	0	0	0	0	1	0	1	0	1	1	1
445	39	0	44	1	2	0	0	105	0	0	1	0	0	1	1	0	0	1	0	0	1	1	0	0	0	0	0	0	0	0	1	0
446	40	0	21	1	2	0	0	105	0	0	1	0	0	0	1	0	0	0	0	0	1	0	0	0	0	0	0	1	0	0	0	1
447	41	0	28	1	4	1	1	105	0	0	1	1	1	0	0	0	0	0	0	0	0	1	0	0	0	0	0	0	0	0	0	0
448	42	0	24	1	4	1	1	105	0	0	1	1	1	0	0	0	0	1	0	0	0	0	0	0	0	0	0	1	0	0	0	0
449	43	0	23	1	3	1	1	105	0	0	1	0	1	0	0	0	0	1	0	0	1	1	0	0	0	0	0	0	1	1	0	0
450	44	1	32	1	3	0	1	105	1	0	0	1	0	0	0	0	0	1	0	0	0	0	0	0	0	0	0	0	0	1	0	0
451	45	1	55	1	4	1	1	105	1	0	0	1	1	0	0	0	0	0	0	0	0	1	0	0	0	0	0	0	1	0	0	0
452	46	1	54	1	3	1	1	105	0	0	1	0	1	0	1	0	0	0	0	0	0	1	0	0	0	0	0	0	0	0	1	0
453	47	0	57	0	3	0	1	105	1	0	0	0	0	0	1	0	0	0	0	0	0	1	0	0	0	0	0	0	1	0	0	0
454	48	1	42	1	2	0	0	105	0	0	1	0	1	1	0	0	0	0	0	0	1	1	0	0	0	0	0	0	0	0	0	0
455	49	0	26	1	3	1	1	105	1	0	0	1	0	0	0	0	0	0	0	0	0	0	0	0	0	0	0	1	0	0	0	0
456	50	0	56	1	3	1	1	105	1	0	0	0	1	0	0	0	0	1	0	0	0	0	0	0	0	0	0	0	0	1	0	0
457	51	1	29	1	4	1	1	105	0	0	1	1	1	0	0	0	0	1	1	1	0	1	0	0	0	0	0	0	1	0	0	1
458	52	0	26	1	4	1	1	105	1	0	0	0	1	0	0	0	0	1	0	0	0	0	0	0	0	0	0	0	1	0	0	0
459	53	0	26	0	2	0	0	105	0	0	1	1	1	0	0	0	0	0	1	0	1	1	1	0	0	0	0	0	1	0	0	0
460	54	0	60	1	5	1	1	105	0	0	1	0	1	0	0	0	0	1	0	0	1	0	0	0	0	0	0	0	1	0	0	0
461	55	0	50	1	3	1	1	105	0	0	1	0	0	1	0	0	0	0	0	0	0	1	0	0	0	0	0	0	1	0	0	0

	A	B	C	D	E	F	G	H	I	J	K	L	M	N	O	P	Q	R	S	T	U	V	W	X	Y	Z	AA	AB	AC	AD	AE	AF
1	**Consumer**	Gender 0F 1M	Age (cont.)	Usual consumer 0 no 1 yes	Freshness 1 not fresh 5 very fresh	will to purchase 0 no 1 yes	**will to consume 0 no 1 yes**	**storage time**	Moisten aspect	Slime	Dry	Bright	Cured aspect	Brownish color	Dull	Darker spots	Greenish spots	Wine aroma	garlic aroma	Fermented aroma	Smoke aroma	Cured aroma	Rancid	Sulfur	Mold	Ammoniac/rotten	Butter	Sour/vinegar aroma	Salty tase	Piquant	Sweet taste	Acid taste
462	56	0	54	1	4	1	1	105	0	0	0	0	1	0	1	0	0	1	1	0	1	1	0	0	0	0	0	0	0	0	0	0
463	57	1	38	1	3	1	1	105	0	0	1	1	0	0	0	0	0	0	0	0	0	0	0	0	0	0	0	1	0	0	0	0
464	58	0	24	1	4	1	1	105	0	0	0	0	0	1	0	1	0	1	0	0	0	0	0	0	0	0	0	1	0	0	0	0
465	59	0	23	1	5	0	0	105	1	0	0	0	1	0	0	0	0	1	0	0	0	0	0	0	0	0	0	0	1	0	0	0
466	60	1	22	1	4	1	1	105	1	0	0	1	1	0	0	0	0	0	0	0	0	1	0	0	0	0	0	0	1	0	0	0
467	61	1	35	1	1	0	0	105	0	0	1	0	1	0	0	1	0	0	0	0	0	1	0	0	0	0	0	0	1	0	0	1
468	62	1	58	1	4	1	1	105	1	0	0	0	0	0	0	0	0	1	0	0	0	1	0	0	0	0	0	0	0	0	0	0
469	63	0	26	1	2	0	0	105	0	0	1	0	1	0	0	0	0	0	0	0	1	1	0	0	0	0	0	0	0	0	0	0
470	64	0	56	1	4	1	1	105	1	0	0	1	1	0	0	0	0	1	0	1	0	0	0	0	0	0	0	0	1	0	0	0
471	65	0	33	1	3	0	1	105	1	0	0	1	0	0	0	0	0	1	0	0	1	0	0	0	0	0	0	0	1	0	0	0
472	66	0	54	1	3	0	0	105	1	0	0	1	1	0	0	0	0	1	0	0	0	1	0	0	0	0	0	0	1	0	0	1
473	67	1	25	1	3	0	1	105	0	0	1	0	0	0	1	0	0	1	1	0	1	0	0	0	0	0	0	1	0	0	0	0
474	68	1	25	1	4	0	1	105	0	0	1	0	0	0	1	0	0	1	1	0	0	0	0	0	0	0	0	0	1	0	0	0
475	69	1	29	1	4	1	1	105	0	0	1	0	1	0	1	0	0	1	1	0	1	1	0	0	0	0	0	0	1	1	0	0
476	70	1	56	1	4	1	1	105	1	0	0	0	0	0	1	0	0	1	0	0	0	0	0	0	0	0	1	0	0	0	0	0
477	71	1	50	1	5	1	1	105	0	0	1	0	0	0	0	0	0	1	0	0	0	0	0	0	0	0	0	0	1	0	0	0
478	72	0	57	1	5	1	1	105	0	0	1	1	1	0	0	0	0	1	1	0	0	1	0	0	0	0	0	1	1	0	0	1
479	73	1	22	1	4	1	1	105	0	0	1	0	1	0	0	0	0	0	0	0	0	1	0	0	0	0	0	0	0	0	1	0
480	74	1	55	1	4	0	1	105	0	0	1	0	0	0	1	0	0	1	1	0	1	0	0	0	0	0	0	1	1	0	0	0
481	75	0	26	1	3	1	1	105	0	0	1	0	1	0	0	0	0	1	0	0	0	1	0	0	0	0	0	0	1	0	0	0

	A	B	C	D	E	F	G	H	I	J	K	L	M	N	O	P	Q	R	S	T	U	V	W	X	Y	Z	AA	AB	AC	AD	AE	AF
1	**Consumer**	Gender 0F 1M	Age (cont.)	Usual consumer 0 no 1 yes	Freshness 1 not fresh 5 very fresh	will to purchase 0 no 1 yes	**will to consume 0 no 1 yes**	**storage time**	Moisten aspect	Slime	Dry	Bright	Cured aspect	Brownish color	Dull	Darker spots	Greenish spots	Wine aroma	garlic aroma	Fermented aroma	Smoke aroma	Cured aroma	Rancid	Sulfur	Mold	Ammoniac/rotten	Butter	Sour/vinegar aroma	Salty tase	Piquant	Sweet taste	Acid taste
482	76	0	53	1	2	0	1	105	0	1	0	0	0	0	0	1	0	1	0	0	1	1	0	0	0	0	0	0	1	0	0	1
483	77	1	53	1	3	1	1	105	0	1	0	0	1	0	0	0	0	1	0	0	0	0	0	0	0	0	0	0	1	0	0	0
484	78	1	58	1	4	1	1	105	0	1	0	0	0	0	1	0	0	1	0	0	0	0	0	0	0	0	0	0	1	0	0	0
485	79	0	60	1	3	0	1	105	0	1	1	0	0	0	0	0	0	0	0	0	0	0	0	0	0	0	0	0	1	0	0	0
486	80	1	26	1	5	1	1	105	0	0	1	1	1	0	0	0	0	1	0	0	0	1	0	0	0	0	0	0	1	0	0	0
487	81	0	27	1	4	0	1	105	0	0	1	0	0	0	1	0	0	1	1	0	1	0	0	0	0	0	0	1	1	0	0	0
488	1	1	24	1	3	0	**0**	126	**0**	0	1	0	1	0	0	0	0	1	1	0	1	1	0	0	0	0	0	1	1	0	0	0
489	2	0	23	1	3	1	1	126	0	0	1	0	1	0	0	0	0	0	0	1	0	1	0	0	0	0	0	0	0	0	0	1
490	3	1	53	1	4	1	1	126	1	0	0	0	1	0	0	0	0	1	1	0	0	0	0	0	0	0	0	0	1	0	0	0
491	4	0	52	1	3	1	1	126	0	0	1	1	1	0	0	0	0	1	1	0	1	0	0	0	0	0	0	0	1	0	0	0
492	5	0	23	1	4	1	1	126	1	0	0	0	1	0	0	0	0	1	1	0	0	1	0	0	0	0	0	1	1	0	0	1
493	6	0	23	1	4	1	1	126	0	0	1	1	0	0	0	1	0	1	1	0	1	1	0	0	0	0	0	0	1	0	0	1
494	7	1	55	1	4	1	1	126	1	0	0	1	1	0	0	0	0	1	1	0	0	1	0	0	0	0	0	0	1	0	0	0
495	8	0	54	1	4	1	1	126	1	0	0	1	1	1	0	0	0	1	1	0	0	0	0	0	0	0	0	0	0	0	0	0
496	9	1	21	1	2	0	0	126	1	1	0	1	0	0	0	1	0	0	0	1	0	1	0	0	0	0	0	1	1	0	0	1
497	10	1	55	1	5	1	1	126	1	0	0	1	1	0	0	0	0	1	0	0	0	1	0	0	0	0	0	1	1	0	0	0
498	11	0	23	0	4	1	1	126	1	0	0	1	0	0	1	0	0	1	0	0	0	1	0	0	0	0	0	0	0	1	0	0
499	12	0	52	1	5	1	1	126	0	0	1	1	1	0	0	0	0	1	1	0	0	1	0	0	0	0	0	0	0	1	0	0
500	13	1	50	0	4	1	1	126	0	0	0	1	1	0	0	0	0	1	1	0	1	1	0	0	0	0	0	0	0	0	0	0
501	14	1	85	1	4	1	1	126	0	0	0	0	0	0	0	0	0	1	1	0	1	1	0	0	0	0	0	0	0	0	0	1

	A	B	C	D	E	F	G	H	I	J	K	L	M	N	O	P	Q	R	S	T	U	V	W	X	Y	Z	AA	AB	AC	AD	AE	AF
1	**Consumer**	Gender 0F 1M	Age (cont.)	Usual consumer 0 no 1 yes	Freshness 1 not fresh 5 very fresh	will to purchase 0 no 1 yes	**will to consume 0 no 1 yes**	**storage time**	Moisten aspect	Slime	Dry	Bright	Cured aspect	Brownish color	Dull	Darker spots	Greenish spots	Wine aroma	garlic aroma	Fermented aroma	Smoke aroma	Cured aroma	Rancid	Sulfur	Mold	Ammoniac/rotten	Butter	Sour/vinegar aroma	Salty tase	Piquant	Sweet taste	Acid taste
502	15	1	60	0	2	0	0	126	0	0	1	1	1	0	0	0	0	0	0	0	0	0	0	0	1	0	0	1	0	0	0	0
503	16	0	58	1	5	1	1	126	0	1	0	1	0	0	0	0	0	0	0	0	0	1	0	0	0	0	0	0	1	0	0	0
504	17	1	30	1	3	1	1	126	0	1	0	0	0	1	0	0	0	0	0	0	0	0	0	0	0	0	0	1	0	0	0	0
505	18	0	59	1	5	1	1	126	1	0	0	1	0	0	0	0	0	1	1	0	1	0	0	0	0	0	0	0	0	0	0	0
506	19	0	23	1	5	0	1	126	0	0	1	0	1	0	0	0	0	0	0	0	0	1	0	0	0	0	0	0	1	0	0	0
507	20	0	22	0	4	1	1	126	0	0	1	0	0	0	1	0	0	1	0	0	0	0	0	0	0	0	0	0	0	0	0	1
508	21	0	23	1	2	0	1	126	0	0	1	0	0	1	1	0	0	1	0	0	0	0	0	0	0	0	0	0	1	0	0	0
509	22	0	28	1	2	0	0	126	1	0	0	1	0	0	0	1	0	0	0	0	0	0	1	0	0	0	0	0	1	0	0	1
510	23	1	23	1	4	1	1	126	1	0	0	0	1	0	0	0	0	0	0	0	0	0	0	0	0	0	0	0	1	0	0	0
511	24	1	39	1	5	1	1	126	1	1	0	1	0	1	0	1	0	0	0	0	0	0	0	0	0	0	0	0	1	0	0	0
512	25	0	26	1	5	1	1	126	0	0	1	1	0	0	0	0	0	0	0	0	0	1	0	0	0	0	0	0	0	0	0	1
513	26	0	27	1	3	0	0	126	0	1	0	1	0	0	0	0	0	1	0	0	0	0	0	0	0	0	1	0	0	0	0	0
514	27	0	36	1	1	0	0	126	0	0	1	1	0	0	0	0	0	0	0	0	0	1	0	0	0	0	0	0	0	0	0	0
515	28	1	49	1	5	0	1	126	1	0	0	0	1	0	0	0	0	0	0	0	0	1	0	0	0	0	0	0	1	0	0	0
516	29	1	23	0	5	1	1	126	0	1	0	1	0	0	0	0	0	1	0	1	0	1	0	0	0	0	0	0	0	0	1	0
517	30	0	46	1	5	1	1	126	1	0	0	0	1	0	0	0	0	1	1	0	0	0	0	0	0	0	0	0	0	0	0	0
518	31	1	47	1	5	1	1	126	1	0	0	1	1	0	0	0	0	0	1	0	1	1	0	0	0	0	0	0	1	0	0	0
519	32	0	53	1	4	1	1	126	0	0	1	0	0	0	0	0	0	1	1	0	0	0	0	0	0	0	0	0	1	0	0	1
520	33	0	48	1	4	1	1	126	0	1	0	1	1	0	0	0	0	0	1	0	0	0	0	0	0	0	0	0	0	0	0	0
521	34	1	52	1	3	1	1	126	1	0	0	1	0	0	0	0	0	0	0	0	1	0	0	0	0	0	0	1	1	0	0	0

	A	B	C	D	E	F	G	H	I	J	K	L	M	N	O	P	Q	R	S	T	U	V	W	X	Y	Z	AA	AB	AC	AD	AE	AF
1	**Consumer**	Gender 0F 1M	Age (cont.)	Usual consumer 0 no 1 yes	Freshness 1 not fresh 5 very fresh	will to purchase 0 no 1 yes	**will to consume 0 no 1 yes**	**storage time**	Moisten aspect	Slime	Dry	Bright	Cured aspect	Brownish color	Dull	Darker spots	Greenish spots	Wine aroma	garlic aroma	Fermented aroma	Smoke aroma	Cured aroma	Rancid	Sulfur	Mold	Ammoniac/rotten	Butter	Sour/vinegar aroma	Salty tase	Piquant	Sweet taste	Acid taste
522	35	0	23	1	5	1	1	126	0	0	1	0	1	0	0	0	0	1	0	0	1	1	0	0	0	0	0	0	1	0	0	0
523	36	0	44	0	4	1	1	126	0	0	1	0	1	0	1	0	0	0	0	0	0	1	0	0	0	0	0	0	0	0	0	0
524	37	0	26	1	3	1	1	126	0	0	1	0	1	0	1	0	0	1	1	0	1	1	0	0	0	0	0	0	1	1	1	0
525	38	0	26	1	1	0	0	126	0	0	1	0	1	0	0	0	0	1	1	0	0	1	0	0	0	0	0	0	1	0	0	0
526	39	0	44	1	2	0	0	126	0	0	1	0	1	0	1	0	0	0	0	0	1	0	0	0	0	0	0	1	0	0	0	1
527	40	0	21	1	2	0	1	126	0	0	1	0	1	0	1	0	0	1	0	0	1	1	0	0	0	0	0	1	0	0	1	0
528	41	0	28	1	1	0	0	126	1	0	0	0	1	0	0	0	0	0	0	0	0	0	0	0	0	0	1	1	0	0	0	0
529	42	0	24	1	4	1	1	126	0	0	1	1	1	0	0	0	0	0	0	0	0	1	0	0	0	0	0	0	0	0	0	0
530	43	0	23	1	4	1	1	126	0	0	1	1	1	0	0	0	0	1	0	0	0	0	0	0	0	0	0	0	0	0	0	0
531	44	1	32	1	3	0	0	126	0	0	1	0	1	0	0	0	0	1	1	0	1	0	0	0	0	0	0	1	1	0	0	0
532	45	1	55	1	3	0	0	126	0	0	1	0	0	0	1	0	0	1	0	0	0	0	0	0	0	0	0	0	0	0	0	1
533	46	1	54	1	4	1	1	126	1	0	0	1	0	0	0	1	0	0	0	0	0	1	0	0	0	0	0	0	0	0	0	0
534	47	0	57	0	4	1	1	126	1	0	0	1	1	0	0	0	0	0	0	1	0	1	0	0	0	0	0	0	0	0	1	0
535	48	1	42	1	1	0	0	126	0	0	1	1	0	0	0	0	0	0	0	1	0	0	0	0	0	0	0	0	0	0	0	0
536	49	0	26	1	3	1	1	126	1	1	0	1	1	1	0	0	0	1	0	0	0	1	0	0	0	0	0	0	1	0	0	0
537	50	0	56	1	3	0	0	126	1	0	0	1	0	0	0	0	0	0	0	0	0	0	0	0	0	0	0	1	0	0	0	0
538	51	1	29	1	2	0	0	126	0	0	0	1	0	0	0	0	0	0	1	0	0	0	0	0	0	0	0	0	0	1	0	0
539	52	0	26	1	4	1	1	126	0	0	1	0	1	0	0	0	0	1	1	1	1	1	0	0	0	0	0	1	1	0	0	1
540	53	0	26	0	4	1	1	126	0	0	1	0	1	0	0	0	0	1	0	0	0	0	0	0	0	0	0	0	1	0	0	0
541	54	0	60	1	4	1	1	126	0	1	0	1	1	0	0	0	0	0	1	0	1	1	0	0	0	0	0	0	1	0	0	0

	A	B	C	D	E	F	G	H	I	J	K	L	M	N	O	P	Q	R	S	T	U	V	W	X	Y	Z	AA	AB	AC	AD	AE	AF
1	**Consumer**	Gender 0F 1M	Age (cont.)	Usual consumer 0 no 1 yes	Freshness 1 not fresh 5 very fresh	will to purchase 0 no 1 yes	**will to consume 0 no 1 yes**	**storage time**	Moisten aspect	Slime	Dry	Bright	Cured aspect	Brownish color	Dull	Darker spots	Greenish spots	Wine aroma	garlic aroma	Fermented aroma	Smoke aroma	Cured aroma	Rancid	Sulfur	Mold	Ammoniac/rotten	Butter	Sour/vinegar aroma	Salty tase	Piquant	Sweet taste	Acid taste
542	55	0	50	1	5	1	1	126	0	1	0	1	1	0	0	0	0	1	0	0	0	0	0	0	0	0	0	0	1	0	0	0
543	56	0	54	1	4	1	1	126	0	1	0	1	0	1	0	0	0	0	0	0	0	1	0	0	0	0	0	0	0	0	0	1
544	57	1	38	1	4	1	1	126	0	0	1	0	1	0	0	0	0	0	0	0	0	0	0	0	0	0	0	1	0	0	0	1
545	58	0	24	1	3	1	1	126	1	0	0	1	0	0	0	0	0	0	1	0	0	0	0	0	0	0	0	0	0	0	0	0
546	59	0	23	1	4	1	1	126	0	0	1	0	0	1	0	0	0	0	0	0	1	0	0	0	0	0	0	0	0	0	0	0
547	60	1	22	1	5	0	0	126	0	0	1	0	1	0	0	0	0	1	0	0	0	0	0	0	0	0	0	0	1	0	0	0
548	61	1	35	1	2	1	1	126	0	0	1	0	0	0	1	0	0	0	0	0	0	0	1	0	0	0	0	0	0	0	0	0
549	62	1	58	1	2	0	0	126	0	0	1	0	0	0	0	1	0	0	0	0	0	0	0	0	0	0	0	0	1	0	0	1
550	63	0	26	1	3	1	1	126	0	0	1	0	1	0	0	0	0	0	1	0	0	0	0	0	0	0	0	0	1	0	0	0
551	64	0	56	1	3	1	1	126	0	0	1	0	0	0	1	0	0	0	1	0	1	0	0	0	0	0	0	0	1	0	0	1
552	65	0	33	1	4	1	1	126	1	0	0	1	1	0	0	0	0	1	1	0	0	1	0	0	0	0	0	0	1	0	0	0
553	66	0	54	1	4	1	1	126	0	0	1	0	0	0	1	0	0	1	1	0	1	0	0	0	0	0	0	0	1	0	0	0
554	67	1	25	1	4	1	1	126	0	0	1	0	0	0	0	1	0	0	0	0	1	0	0	0	0	0	0	0	0	0	0	1
555	68	1	25	1	5	1	1	126	0	0	1	1	1	0	0	0	0	1	1	0	0	1	0	0	0	0	0	0	1	0	0	1
556	69	1	29	1	5	1	1	126	0	0	1	1	1	0	0	0	0	1	1	0	0	0	0	0	0	0	0	0	1	0	0	0
557	70	1	56	1	3	1	1	126	0	0	1	0	1	0	0	0	0	0	0	0	1	0	0	0	0	0	0	0	0	1	0	0
558	71	1	50	1	5	1	1	126	1	0	0	0	1	0	0	0	0	1	0	0	0	0	0	0	0	0	0	1	1	0	0	0
559	72	0	57	1	4	0	0	126	0	1	0	0	1	0	1	1	0	1	0	0	0	1	0	0	0	0	0	1	0	0	0	0
560	73	1	22	1	1	0	1	126	0	0	1	0	1	0	1	1	0	1	1	0	0	0	0	0	0	0	0	0	1	0	0	0
561	74	1	55	1	4	0	1	126	0	0	1	0	1	0	1	0	0	1	1	0	1	1	0	0	0	0	0	0	1	1	0	0

	A	B	C	D	E	F	G	H	I	J	K	L	M	N	O	P	Q	R	S	T	U	V	W	X	Y	Z	AA	AB	AC	AD	AE	AF
1	**Consumer**	Gender 0F 1M	Age (cont.)	Usual consumer 0 no 1 yes	Freshness 1 not fresh 5 very fresh	will to purchase 0 no 1 yes	**will to consume 0 no 1 yes**	**storage time**	Moisten aspect	Slime	Dry	Bright	Cured aspect	Brownish color	Dull	Darker spots	Greenish spots	Wine aroma	garlic aroma	Fermented aroma	Smoke aroma	Cured aroma	Rancid	Sulfur	Mold	Ammoniac/rotten	Butter	Sour/vinegar aroma	Salty tase	Piquant	Sweet taste	Acid taste
562	75	0	26	1	3	1	1	126	1	0	0	1	1	0	0	0	0	0	0	0	0	1	0	0	0	0	0	1	1	0	0	0
563	76	0	53	1	3	1	1	126	0	0	0	0	1	0	0	0	0	0	0	0	1	0	0	0	0	0	0	0	0	1	0	0
564	77	1	53	1	4	1	1	126	0	0	1	0	1	0	0	0	0	1	0	0	0	1	0	0	0	0	0	0	1	1	0	0
565	78	1	58	1	3	0	1	126	1	0	0	0	0	1	0	0	0	1	0	0	1	0	0	0	0	0	0	0	1	0	0	0
566	79	0	60	1	4	1	1	126	0	1	0	0	0	0	1	0	0	1	0	0	0	0	0	0	0	0	0	0	1	0	0	0
567	80	1	26	1	3	0	1	126	0	0	1	0	1	0	0	0	0	0	0	0	0	0	0	0	0	0	0	0	1	0	0	0
568	81	0	27	1	5	1	1	126	0	0	1	0	1	0	0	0	0	1	0	0	0	1	0	0	0	0	0	0	1	0	0	0
